# Auxin Response Factor 2A Is Part of the Regulatory Network Mediating Fruit Ripening Through Auxin-Ethylene Crosstalk in Durian

**DOI:** 10.3389/fpls.2020.543747

**Published:** 2020-09-09

**Authors:** Gholamreza Khaksar, Supaart Sirikantaramas

**Affiliations:** ^1^ Molecular Crop Research Unit, Department of Biochemistry, Faculty of Science, Chulalongkorn University, Bangkok, Thailand; ^2^ Omics Sciences and Bioinformatics Center, Chulalongkorn University, Bangkok, Thailand

**Keywords:** auxin, auxin response factor, durian, ethylene, fruit ripening, transcriptional regulation

## Abstract

Fruit ripening is a highly coordinated developmental process driven by a complex hormonal network. Ethylene is the main regulator of climacteric fruit ripening. However, a putative role of other key phytohormones in this process cannot be excluded. We previously observed an increasing level of auxin during the post-harvest ripening of the durian fruit, which occurred concomitantly with the rise in the climacteric ethylene biosynthesis. Herein, we connect the key auxin signaling component, auxin response factors (ARFs), with the regulatory network that controls fruit ripening in durian through the identification and functional characterization of a candidate ripening-associated ARF. Our transcriptome-wide analysis identified 15 ARF members in durian (*DzARF*s), out of which 12 were expressed in the fruit pulp. Most of these *DzARF*s showed a differential expression, but *DzARF2A* had a marked ripening-associated expression pattern during post-harvest ripening in Monthong, a commercial durian cultivar from Thailand. Phylogenetic analysis of DzARF2A based on its tomato orthologue predicted a role in ripening through the regulation of ethylene biosynthesis. Transient expression of *DzARF2A* in *Nicotiana benthamiana* leaves significantly upregulated the expression levels of ethylene biosynthetic genes, pointing to a ripening-associated role of DzARF2A through the transcriptional regulation of ethylene biosynthesis. Dual-luciferase reporter assay determined that DzARF2A trans-activates durian ethylene biosynthetic genes. We previously reported significantly higher auxin level during post-harvest ripening in a fast-ripening cultivar (Chanee) compared to a slow-ripening one (Monthong). *DzARF2A* expression was significantly higher during post-harvest ripening in the fast-ripening cultivars (Chanee and Phuangmanee) compared to that of the slow-ripening ones (Monthong and Kanyao). Thus, higher auxin level could upregulate the expression of *DzARF2A* during ripening of a fast-ripening cultivar. The auxin-induced expression of *DzARF2A* confirmed its responsiveness to exogenous auxin treatment in a dose-dependent manner, suggesting an auxin-mediated role of DzARF2A in fruit ripening. We suggest that high *DzARF2A* expression would activate ARF2A-mediated transcription of ethylene biosynthetic genes, leading to increased climacteric ethylene biosynthesis (auxin-ethylene crosstalk) and faster ripening. Hence, we demonstrated DzARF2A as a new component of the regulatory network possibly mediating durian fruit ripening through transcriptional regulation of ethylene biosynthetic genes.

## Introduction

Fruit ripening is a genetically programmed and coordinated developmental process involving dramatic physiological transformations, some of which include changes in the aroma, texture, color, and nutritional value of the flesh (Alexander and Grierson, 2002; Adams-Phillips et al., 2004). This process is controlled by transcriptional regulatory networks with transcription factors (TFs) acting as pivotal regulators. Depending on the mechanism, the ripening of fleshy fruits can be divided into climacteric and non-climacteric ripening (Lelievre et al., 1997). Climacteric ripening is associated with an autocatalytic increase in ethylene biosynthesis and the key role played by ethylene in this process (as the main trigger of fruit ripening) has been well documented (Barry et al., 2000). Consistently, various genes involved in ethylene biosynthesis and signaling were shown to be vital for ripening (Ayub et al., 1996; Xie et al., 2006; Wang et al., 2009), but the involvement of other plant hormones in this process (together with ethylene) seems also likely. The possibility that fruit ripening is regulated by a complex hormonal network has already been formulated and suggested by the existing literature. Auxin (IAA; indole-3-acetic acid) is a crucial phytohormone which regulates numerous aspects of plant growth and development (Woodward and Bartel, 2005), including ripening (Jones et al., 2002). Along with significant increases in the climacteric ethylene, elevated auxin levels of have also been detected during the ripening of tomato (Gillaspy et al., 1993), peach (Miller et al., 1987), and durian (Khaksar et al., 2019), suggesting a possible ripening-associated role of auxin. Auxin response factor (ARF) TFs represent the core of auxin signaling. These proteins mediate the auxin-dependent gene expression *via* binding to the auxin response elements (AuxREs; TGTCTC), located in the promoter regions of the auxin responsive genes, in a dose-dependent manner (Hagen and Guilfoyle, 2002). ARFs are generally characterized by a highly conserved DNA binding domain (DBD) at their N terminus. They also contain a middle region, responsible for the transactivation or repression of target genes and a carboxyl-terminal dimerization domain (CTD) that is involved in the protein-protein interactions (Guilfoyle and Hagen, 2007; Guilfoyle and Hagen, 2012; Nanao et al., 2014; Guilfoyle, 2015). The domain architecture of ARFs is known to play a key role in their specificity. Since the identification and characterization of the first Arabidopsis *ARF* (*AtARF1*) (Ulmasov et al., 1997), numerous studies have identified members of the ARF gene family in plant species, such as tomato (Kumar et al., 2011), rice (Wang et al., 2007), Medicago (Shen et al., 2015), banana (Hu et al., 2015), apple (Luo et al., 2014), maize (Xing et al., 2011), chickpea (Singh et al., 2017), physic nut (Tang et al., 2018), grape (Wan et al., 2014), and papaya (Liu et al., 2015). Functional characterization of some ARFs based on the phenotypes of the loss-of-function and gain-of-function mutants have also been carried out. For instance, the Arabidopsis *arf1* and *arf2* mutants showed abnormal abscission of the floral organs (Ellis et al., 2005), while mutation in the *AtARF7* gene diminished hypocotyl response to blue light and auxin (Harper et al., 2000). The *AtARF8* loss-of-function mutation, on the other hand, impaired hypocotyl elongation and auxin homeostasis (Goetz et al., 2006), while the tomato *SlARF3* was found to participate in the formation of trichomes and epidermal cells (Zhang et al., 2015). In addition to their roles in plant growth and development, ARFs are also involved in the responses to abiotic stressors. *OsARF16*, for example, has been found to participate in the adaptive response to phosphate starvation in rice (Shen et al., 2013), while another member (*OsARF12*) has been found to play role in iron homeostasis (Qi et al., 2013). Although the links between ARFs and plant growth and development have been extensively studied, our actual knowledge regarding the possible roles of ARFs in fruit ripening remains poorly understood and limited only to a few studies in tomato and papaya. In tomato, the *SlARF2A* and *SlARF2B* (two *ARF2* paralogs) displayed a ripening-associated expression pattern. Down-regulation of either *SlARF2A* or *SlARF2B* led to ripening defects, while the *arf2a/arf2b* double mutation resulted in a severe inhibition of the ripening process (Hao et al., 2015). Moreover, a significant decrease in the climacteric ethylene biosynthesis and the expression levels of ethylene biosynthetic genes was observed in the *SlARF2AB-RNAi* fruits (Hao et al., 2015). Breitel et al. (2016) identified *SlARF2A*, an auxin signaling component that controls ripening. They found *SlARF2A* expression to be ripening-regulated and responsive to exogenous ethylene and auxin treatments. In papaya, Liu et al. (2015) profiled the expression pattern of 11 *ARF*s (*CpARF*s) during the different fruit developmental stages and found that the expression of most *CpARF*s underwent significant changes with ripening. Specifically, the expression of *CpARF1* and *CpARF2* increased, while that of *CpARF7* and *CpARF11* decreased.

Durian (*Durio zibethinus* Murr.) is a tropical fruit crop of rising economy value endemic to Southeast Asia, with an ever-growing popularity on the international market. Durian is distinctive for its strong and unique odor and formidable spiny husk. With over 200 existing cultivars, Thailand is the top durian exporter across the Southeast Asian region. However, only a few of these cultivars are commercially cultivated and in high demand both locally and on the international market. These include Monthong (*D. zibethinus* Murr. cv. “Monthong”), Chanee (*D. zibethinus* Murr. cv. “Chanee”), Phuangmanee (*D. zibethinus* Murr. cv. “Phuangmanee”), and Kanyao (*D. zibethinus* Murr. cv. “Kanyao”). Among these, Monthong—due to its mild odor and creamy texture—is of great interest (Pinsorn et al., 2018). Notably, these cultivars harbor different post-harvest ripening behaviors; Monthong and Kanyao are slow-ripening cultivars that need around 5 days to fully ripen after being harvested at mature stage. Chanee and Phuangmanee, on the other hand, are fast-ripening cultivars that ripen 3 days after harvesting at mature stage. Durian is a climacteric fruit that has a strikingly limited shelf life after harvesting. Hence, the fast and slow ripening processes are agronomic traits of great economic importance for agricultural industry. Offering durian fruit with a pleasant flavor and nutritional value, while also harboring longer shelf life remains a challenge for the industry. Over recent decades, investigations have focused on extending shelf life. However, our actual knowledge on the molecular mechanisms underlying the ripening process is still limited, especially with regard to fast- and slow-ripening cultivars. In our previous study, we observed an increasing level of auxin production during the post-harvest ripening of the durian fruit in both Monthong and Chanee cultivars, suggesting a ripening-associated role of auxin during fruit ripening (Khaksar et al., 2019). Here, considering the major role of ARFs in the auxin signaling pathway, we performed a transcriptome-wide analysis on the durian genome and identified 15 members of the ARF gene family. Comprehensive gene expression profiling of the durian *ARF*s (*DzARF*s) during the post-harvest ripening revealed significant differential expression of some *ARF*s and suggested putative ripening-associated ones. We then functionally characterized a candidate ripening-associated TF (DzAFR2A). Our findings positioned DzARF2A as a key component of the regulatory network behind the post-harvest ripening of the durian. To our knowledge, this is the first report on the identification and characterization of the ARF gene family in this fruit. Findings from this study can be further exploited towards the development of molecular markers for breeding new durian varieties with longer shelf lives.

## Materials and Methods

### Plant Materials and Treatments

Durian (*Durio zibethinus* Murr.) cv. Monthong, Chanee, and Phuangmanee were collected from commercial durian plantations located in the eastern part of Thailand. Fruit samples having similar size and weight (~3–4 kg each) were harvested at mature stage which occurs at 90 days (for Chanee and Phuangmanee) and 105 days (for Monthong) after anthesis. Samples were kept at room temperature (30°C) until peeling. For these cultivars, three types of samples during post-harvest ripening (unripe, midripe, and ripe) were used. For unripe samples, fruits harvested at the mature stage of all three cultivars were kept at room temperature for one day and then peeled. For midripe samples, fruits harvested at the mature stage were kept at room temperature for two days (for Chanee and Phuangmanee) and three days (for Monthong) and then peeled. For ripe samples, fruits harvested at the mature stage were kept at room temperature for three days (for Chanee and Phuangmanee) and five days (for Monthong) and then peeled. In this study, another cultivar called Kanyao was also used. However, for Kanyao cultivar, we only had access to the ripe fruit which was bought at the ripe stage and peeled. After peeling, two of the central pulps were collected from each fruit sample, following the method described in Pinsorn et al. (2018). To ensure samples of the different cultivars were compared at the same ripening stage, the first pulp was collected along with a seed, and was used to measure fruit firmness with a texture analyzer following our previous study (Khaksar et al., 2019). Midripe and ripe pulps had a mean [± standard deviation (SD)] firmness of 3.4 ± 0.81 and 1.55 ± 0.45 N, respectively in all cultivars. After this test, the second pulp was collected without a seed, immediately frozen in liquid nitrogen, and stored at -80°C for RNA extraction.

To further validate the putative ripening-associated *DzARF*s in Monthong cultivar, three different ripening treatments were applied as follows: natural, ethephon-induced, and 1-methylcyclopropene (1-MCP)-delayed ripening. Durian samples were collected at mature stage and treated with either ethephon (48% 2-chloroethylphosphonic acid; Alpha Agro Tech Co., Ltd., Thailand) or 1-MCP (0.19% 1-MCP tablet; BioLene Co., Ltd., China) for ethephon-induced and 1-MCP-delayed ripening, respectively following the method described in our previous study (Khaksar et al., 2019). After treatments, control and treated samples were kept at room temperature (30°C) until the ethephon-induced samples ripened. As soon as ethephon-treated samples ripened, all samples from the other ripening conditions were peeled. Two central pulps were collected from each sample and processed as mentioned previously. In this study, for each type of sample (unripe, midripe, and ripe, as well as natural ripening, ethephon-induced ripening, and 1-MCP-delayed ripening) of each cultivar, three independent biological replicates were used. Each biological replicate is one durian fruit harvested from a separate tree.

For transient expression of *DzAFR2A* in *Nicotiana benthamiana* leaves, *N. benthamiana* seeds were sown in pots containing peat moss and were grown under controlled conditions (temperature 25°C and 16/8 h light/dark photoperiod; artificial light of 4,500 Lux). Two-week-old seedlings were transplanted individually into pots and were grown under similar conditions.

For exogenous auxin treatment, young leaves of Monthong cultivar were soaked in different concentrations (10, 20, and 40 μM) of indole-3-acetic acid (IAA) (Duchefa Biochemie, The Netherlands) for 1 and 2 h. As control, leaves were soaked in distilled water without IAA. For reverse transcription quantitative polymerase chain reaction (RT-qPCR) analysis, leaves were immediately frozen in liquid nitrogen and stored at -80°C until total RNA extraction.

### Transcriptome-Wide Identification and Tissue-Specific Expression Analysis of Durian *ARF*s

In order to obtain the assembled transcriptome data, Illumina sequencing reads from RNA-Seq study of durian (Musang King cultivar) were retrieved from a public repository database (SRA, Sequence Read Archive) with the following accession numbers: SRX3188225 (root tissue), SRX3188222 (stem tissue), SRX3188226 (leaf tissue), and SRX3188223 (aril/pulp tissue). To obtain the *de novo* assembled transcriptome, we followed the method described in our previous study (Khaksar et al., 2019). We then followed Hidden Markov Model (HMM) search to identify ARF gene family members. First, we obtained the ARF conserved domain (PF06507) from the Pfam protein database (http://pfam.xfam.org) and used as a query to search against the assembled transcriptome database using the HMMER software (http://hmmer.org/) following e-value cut-off ≤1e^-5^. This approach enabled us to identify 15 non-redundant ARF gene family members. Further, the amino acid sequences of durian ARFs (DzARFs) were checked in SMART database for the presence of the ARF domain. The expression levels of *DzARF*s in different tissues, including root, stem, leaf, and aril (pulp) were analyzed using the *de novo* assembled transcriptome. Abundance estimation tool in Trinity package was used to align the input reads to the *de novo* assembled transcriptome to obtain raw counts of each contig. The raw read counts were then merged in a single read count matrix which was then normalized to generate a Trimmed Mean of M-values (TMM) normalized matrix. For generating heatmap, the normalized total read counts were used as queries in MetaboAnalyst 4.0, an open source R-based program (Chong et al., 2018). For the heatmap construction, the values were sum normalized, log2 transformed and autoscaled.

### Phylogenetic Analysis

To study evolutionary relationships among DzARFs and ARFs from Arabidopsis and tomato, a neighbor-joining (NJ) phylogenetic tree was constructed with 1,000 bootstrap replicates using MEGA X software (with a JTT model and pairwise gap deletion) by aligning their full-length protein sequences (Kumar et al., 2018).

### Prediction of Conserved Motifs and Gene Structure

MEME program (http://meme-suite.org) was used for identification of the conserved motifs (Bailey et al., 2009) with the following parameter settings: motif length = 6–100; motif sites = 2–120; maximum number of motifs = 10; and the distribution of one single motif was “any number of repetitions”. Exon/intron organization of the durian ARF genes was determined using Gene Structure Display Server 2.0 (with default parameters) (http://gsds.cbi.pku.edu.cn).

### RT-qPCR Analysis

Total RNA isolation from durian fruit pulp samples was carried out using PureLink Plant RNA Reagent (Thermo Fisher Scientific™) following the manufacturer’s instructions. Genomic DNA was removed with DNase I (Thermo Fisher Scientific™) treatment. The quality and quantity of RNA samples were examined using agarose gel electrophoresis and an Eppendorf BioPhotometer D30 with A260/280 and A260/230 ratios between 1.8 to 2.0 and 2.0 to 2.2, respectively following the standard guidelines described in Bustin et al. (2009). For reverse transcription, one microgram of total RNA was used to generate cDNA using a RevertAid First Strand cDNA Synthesis Kit (Thermo Fisher Scientific™), following the manufacturer’s recommended protocol and the standard guidelines of reverse transcription described in Bustin et al. (2009). Primer 3 online (http://primer3.ut.ee/) was used to design the primers used in this study which are presented in **Supplementary Table S1**. To measure the transcript levels of *DzARF*s at three stages (unripe, midripe, and ripe) during post-harvest ripening of Monthong and compare the expression level of candidate ripening-associated *DzARF* among different cultivars, RT-qPCR was performed. The PCR reaction was performed in a total volume of 10 μl containing 1 μl of diluted cDNA (1 ng of cDNA), 5 μl of 2x QPCR Green Master Mix LRox (biotechrabbit, Berlin, Germany), and 200 nM of each gene-specific primer. A CFX95 Real-time System (Bio-Rad Laboratories Inc., California, USA) was used under the following conditions: initial activation at 95°C for 3 min, followed by 40 cycles of denaturation at 95°C for 15 s, then annealing at 58–60°C for 30 s, and finally extension at 72°C for 20 s. Three independent biological replicates were used for each RT-qPCR experiment. The elongation factor 1 alpha (*EF-1α*; XM_022889169.1) and actin (*ACT*; XM_022897008.1) genes of durian were selected as reference genes for the normalization of RT-qPCR data according to our in-house transcriptome data of durian fruit from different cultivars which confirmed the invariant expression levels of *EF-1α* and *ACT* under different experimental conditions. In addition, to further confirm this, we used Genorm software (Vandesompele et al., 2002) to check the stability of our reference genes (see **Supplementary Table S1** for primers).

Statistical analysis of RT-qPCR data was carried out using the method described in Steibel et al. (2009) which consists of the analysis of cycles to threshold values (Ct) for the target and reference genes according to a linear mixed model [Model I of the report of Steibel et al. (2009)]. Treatments, genes, and their interactions were the fixed factors whereas the obtained Ct values comprised the dependent variables of the model. Samples were the random effects of the model. The normalization of RT-qPCR expression data was carried out using normalization factors; the geometric mean of the two reference genes calculated with Genorm software (Vandesompele et al., 2002). For graphical illustration, the expression data are presented as fold change (2^-ΔΔCt^) (Livak and Schmittgen, 2001). *P* < 0.05 was considered statistically significant. R programming language (https://www.R-project.org/) was used for the analysis.

### Transient Expression of *DzARF2A* in *N. benthamiana* Leaf

The full-length cDNA of *DzARF2A* was amplified from the cDNA of Monthong cultivar (at midripe stage) using Phusion Hot Start II High-Fidelity DNA Polymerase (Thermo Fisher Scientific, Waltham, MA, USA) (primers are listed in **Supplementary Table S2**). After that, the PCR amplicon was inserted into the entry vector pDONR207 using Gateway^®^ BP Clonase^®^ II (Thermo Fisher Scientific, Waltham, MA, USA). After sequencing, *DzARF2A* in Monthong cultivar was confirmed to be identical to the *DzARF2A* in Musang King cultivar (XM_022883347.1). From the resultant pDONR207-*DzARF2A*, the putative *DzARF2A* was inserted in to the pGWB2 expression vector using Gateway^®^ LR Clonase^®^ II (Thermo Fisher Scientific, Waltham, MA, USA) to produce pGWB2-*DzARF2A*. The resulting pGWB2-*DzARF2A* as well as pGWB2-*GFP* (control) were individually introduced into the *Agrobacterium tumefaciens* strain GV3101 by electroporation. The positive *A. tumefaciens* colony harbouring each construct was further grown in 25 ml of LB broth containing 25 mg L^-1^ gentamycin and 25 mg L^-1^ rifampicin with shaking at 250 rpm overnight at 30°C. Cells were harvested by centrifugation at 4,000×g for 10 min and resuspended in MM buffer (10 mM MES and 10 mM MgCl_2_, pH 5.6) to reach an optical density of 0.6 (OD_600_). For the agroinfiltration experiment, the agrobacterium solution containing either pGWB2-*DzARF2A* or pGWB2-*GFP* (control) was mixed with the agrobacterium solution harbouring the gene encoding the silencing inhibitor protein p19 at a ratio of 1:1. Then, acetosyringone was added at a concentration of 100 mg L^-1^ and the mixture was incubated at room temperature for 3 h under dark conditions. Then, the solution was infiltrated into the abaxial surface of three individual leaves per plant using a needleless 1 ml syringe. Three independent biological replicates (three 4-week-old plants) were used for each construct at each timepoint. At three and five days after infiltration, samples of the infiltrated leaves were collected, frozen in liquid nitrogen and ground into a fine powder with a mortar and pestle. This powder was then used for RNA isolation and cDNA synthesis. The cDNA was further used as the template for RT-qPCR analysis. The elongation factor 1 alpha (*EF-1α*; XM_016613715.1) and actin (*ACT*; XM_016618072.1) genes of *Nicotiana* were used as reference genes for the normalization of RT-qPCR data. In order to design primers for *N. benthamiana* ethylene biosynthetic genes, the *Nicotiana tabacum* genome [i.e., 1-aminocyclopropane-1-carboxylic acid (ACC) synthase; *ACS* (NM_001326261.2) and ACC oxidase; *ACO* (NM_001325823.1)] were used to blast against the genome sequence of *N. benthamiana* (SGN *Nicotiana benthamiana* ftp site; https://solgenomics.net/organism/Nicotiana_benthamiana/genome). Primers are listed in **Supplementary Table S2**. The 2000-bp upstream promoter regions of these genes were scanned for the auxin response elements (AuxREs; TGTCTC) using the online tool PLACE (http://www.dna.affrc.go.jp/) (Liu et al., 2017).

### Dual-Luciferase Reporter Assay

To examine the binding activity of DzARF2A to the promoters of ethylene biosynthetic genes, dual-luciferase assay was performed following the method described in Feng et al. (2016). Briefly, genomic DNA was extracted from durian leaves using the cetyltrimethyl ammonium bromide (CTAB)-based method according to Abdel-Latif and Osman (2017). The 2,000-bp promoter regions of ethylene biosynthetic genes of durian; *DzACS* (XM_022901720.1) and *DzACO* (XM_022903265.1) were amplified using the primers listed in **Supplementary Table S2** and cloned into the pGreenII 0800-LUC double-reporter vector (Hellens et al., 2005). For generating the effector construct, the coding sequence of *DzARF2A* was cloned into the pGWB2 expression vector. *A. tumefaciens* (GV3101) harbouring each of the reporter or effector constructs were then co-infiltrated into *N*. *benthamiana* leaves. After three days, firefly (LUC) and *Renilla* (REN) luciferase activities were measured by a dual-luciferase reporter assay system (Promega, E1910) according to the manufacturer’s instructions on a Tecan Infinite 200 PRO microplate reader (TECAN) and the relative LUC/REN ratios were calculated. Four independent biological replicates were used.

## Results

### Transcriptome-Wide Identification of *ARF*s in Durian

Based on the bioinformatics analysis of the publicly available transcriptomic data from different tissues (stem, root, leaf, and pulp) of the durian cv. Musang King, we identified 15 genes encoding putative ARFs (**Table 1**). The deduced DzARFs ranged from 576 (ARF17) to 1142 (ARF7) amino acids in length and varied from 63.65 (ARF17) to 128.00 (ARF7) kDa in molecular weight. Their isoelectric points were between 5.10 (ARF5A) and 8.50 (ARF10), suggesting that they could function in various microenvironments.

**Table 1:** List of *DzARF* genes identified by a transcriptome-wide analysis.

Gene name	ORF length (bp)	Protein ID	Protein length (aa)	Molecular weight (KDa)	Isoelectric point (pI)
ARF1	2010	XP_022716380.1	669	74.24	5.64
ARF2A	2556	XP_022739082.1	851	94.35	6.11
ARF2B	2136	XP_022758097.1	711	80.24	7.55
ARF3	2211	XP_022724470.1	736	80.35	6.65
ARF4	2391	XP_022752446.1	796	88.04	6.33
ARF5A	2691	XP_022772671.1	896	98.90	5.10
ARF5B	2844	XP_022769144.1	947	104.32	5.21
ARF6	2706	XP_022720240.1	901	99.72	6.10
ARF7	3429	XP_022732351.1	1142	128.00	6.08
ARF8	2406	XP_022759400.1	801	88.88	5.92
ARF9	2097	XP_022721637.1	698	77.90	6.10
ARF10	2133	XP_022730470.1	710	78.29	8.50
ARF17	1731	XP_022746581.1	576	63.65	5.47
ARF18	2127	XP_022752863.1	708	77.77	8.46
ARF19	3342	XP_022729004.1	1113	123.10	6.25

### Phylogenetic Analysis

**Figure 1:**
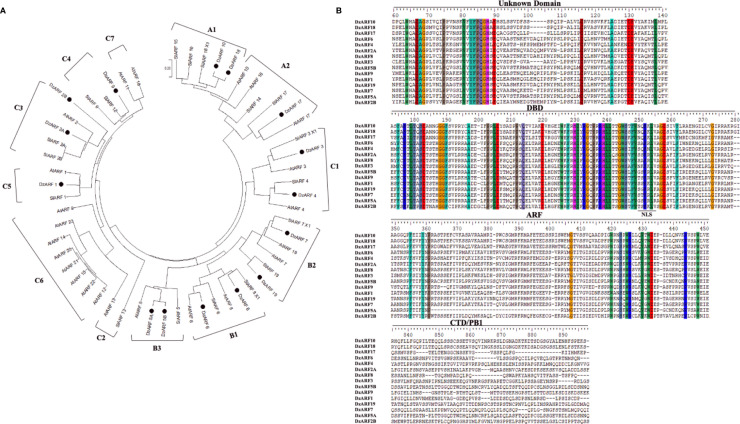
Phylogenetic tree and multiple sequence alignment of the amino acid sequences of the DzARFs. **(A)** The deduced full-length protein sequences of durian ARFs (DzARFs) were aligned with *Arabidopsis thaliana* (AtARFs) and *Solanum lycopersicum* (SlARFs) protein sequences to construct the phylogenetic tree using MEGA X software and the neighbor-joining method (with 1,000 bootstrap replicates, a JTT model and pairwise gap deletion using a bootstrap test of phylogeny with the minimum evolution test and default parameters). **(B)** Multiple sequence alignment analysis was carried out using ClutalW. Identical amino acids are highlighted by color. Only important domains are presented (DBD, DNA binding domain; ARF, auxin response factor domain; CTD/PB1, carboxyl-terminal dimerization/Phox and Bem 1 domain). A nuclear localization signal (NLS) was identified at the end of DBD in all DzARFs.

To examine the evolutionary relationships between DzARFs and the previously-reported ARFs from the model plants Arabidopsis and tomato, we constructed a NJ phylogenetic tree based on the full-length amino acid sequence similarity and topology of 23 Arabidopsis ARFs (AtARFs) and 18 tomato ARFs (SlARFs), by aligning their full-length protein sequences with 1,000 bootstrap replicates using MEGA X. Our phylogenetic analysis classified DzARFs into three major clades (A, B, and C), which were further classified into 12 subclades (A1, A2, B1, B2, B3, C1, C2, C3, C4, C5, C6, and C7) (**Figure 1A**). Subclades C2, C6, and C7 lacked any DzARFs and harbored AtARFs and/or SlARFs. Group C6 was the biggest subclade and was comprised of eight members, whereas groups C2 and C7 were the smallest ones and harbored only two members in each group.

### Multiple Sequence Alignment, Determination of Conserved Motifs and Gene Structure Analysis

Multiple peptide sequence alignment of DzARFs revealed a highly conserved DBD of around 80 amino acids at the N-terminal region (**Figure 1B**). Moreover, these DzARFs contained a middle region that functions as a transcriptional activation or repression domain and a CTD responsible for the protein-protein interactions. Notably, we also identified an unknown domain located at the early N-terminal region of the DzARF protein sequences (**Figure 1B**). Moreover, our *in silico* analyses revealed the presence of a conserved putative mono-partite nuclear localization signal (NLS) at the end of the DBD of all DzARFs which confirmed their localization to the nucleus (**Figure 1B**). Our observation is in line with previous studies which have well documented the occurrence of NLS in members of the ARF TF family (Shen et al., 2010; Yu et al., 2014; Niu et al., 2018; Diao et al., 2020).

By using MEME, we analyzed the protein sequence features of DzARFs and identified at least 10 conserved motifs (**Supplementary Figure S1**). Among these, two novel motifs (1 and 2) were associated with the N-terminal unknown domain. Motifs 3, 4, and 5 were found to be associated with the DBD. The ARF domain corresponded to motifs 6, 7, and 8 and the CTD corresponded to motifs 9 and 10. Notably, the DzARFs in the phylogenetic tree harbored similar motif organization and clustered together (**Supplementary Figure S1**). We also analyzed the exon-intron organization of *DzARF*s and found no introns within the *DzARF*s (**Supplementary Figure S2**). This observation was similar to members of the Dof gene family in durian (*DzDof*s), which harbored no introns (Khaksar et al., 2019).

### Tissue-Specific Expression of *DzARF*s

**Figure 2:**
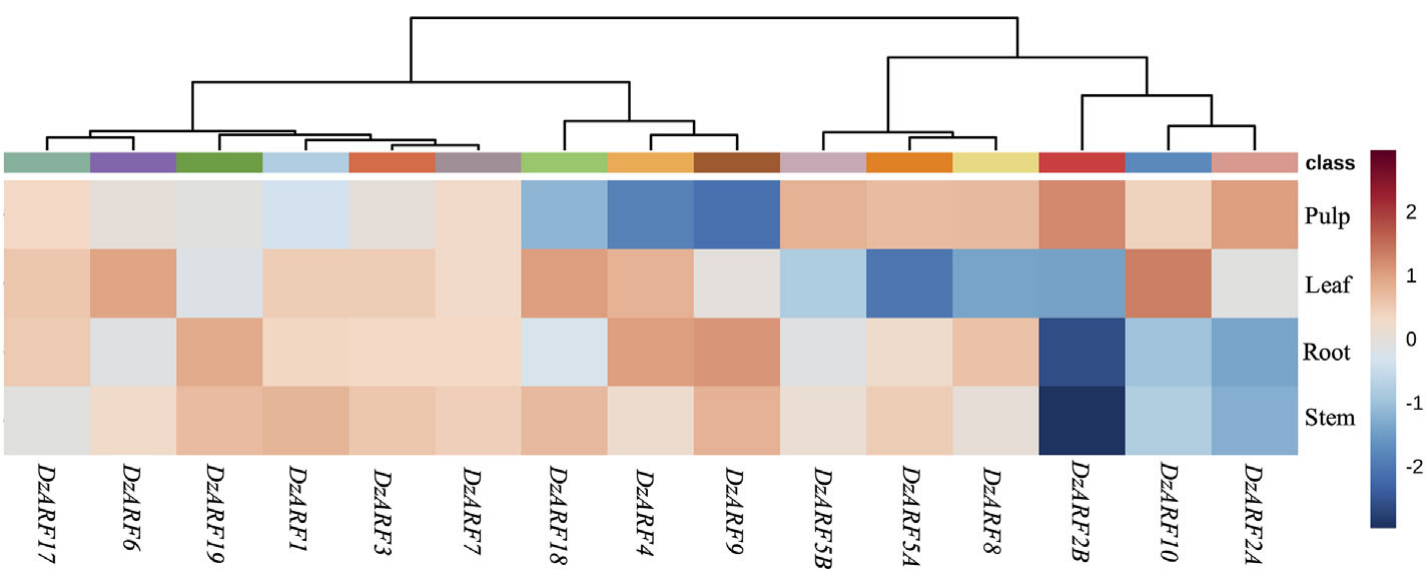
Tissue-specific expression profile of *DzARF*s in Musang King cultivar at ripe stage. The publicly available Illumina RNA-seq data were used to analyze the expression levels of *DzARF*s in root, stem, leaf, and fruit pulp tissues. The higher expression for each gene was presented in red; otherwise, blue was used. Heatmap was generated using MetaboAnalyst 4.0, an open source R-based program. Data were sum normalized, log transformed, and auto scaled.

We analyzed the publicly available transcriptomic data on the different tissues (stem, root, leaf, and pulp) from durian cv. Musang King and observed that 12 out of the 15 *DzARF*s (*DzARF1*, *DzARF2A*, *DzARF2B*, *DzARF3*, *DzARF5A*, *DzARF5B*, *DzARF6*, *DzARF7*, *DzARF8*, *DzARF10*, *DzARF17*, and *DzARF19*) were expressed in the fruit pulp (**Figure 2**). This suggests a possible role of these *DzARF*s in the post-harvest ripening of the durian fruit. The three remaining *DzARF*s, including *DzARF4*, *DzARF9*, and *DzARF18* were expressed in other tissues, but not in the fruit pulp (**Figure 2**), suggesting that their roles in fruit ripening seem unlikely.

### Differential Expression of the *DzARF*s During Post-Harvest Ripening of Durian cv. Monthong

**Figure 3:**
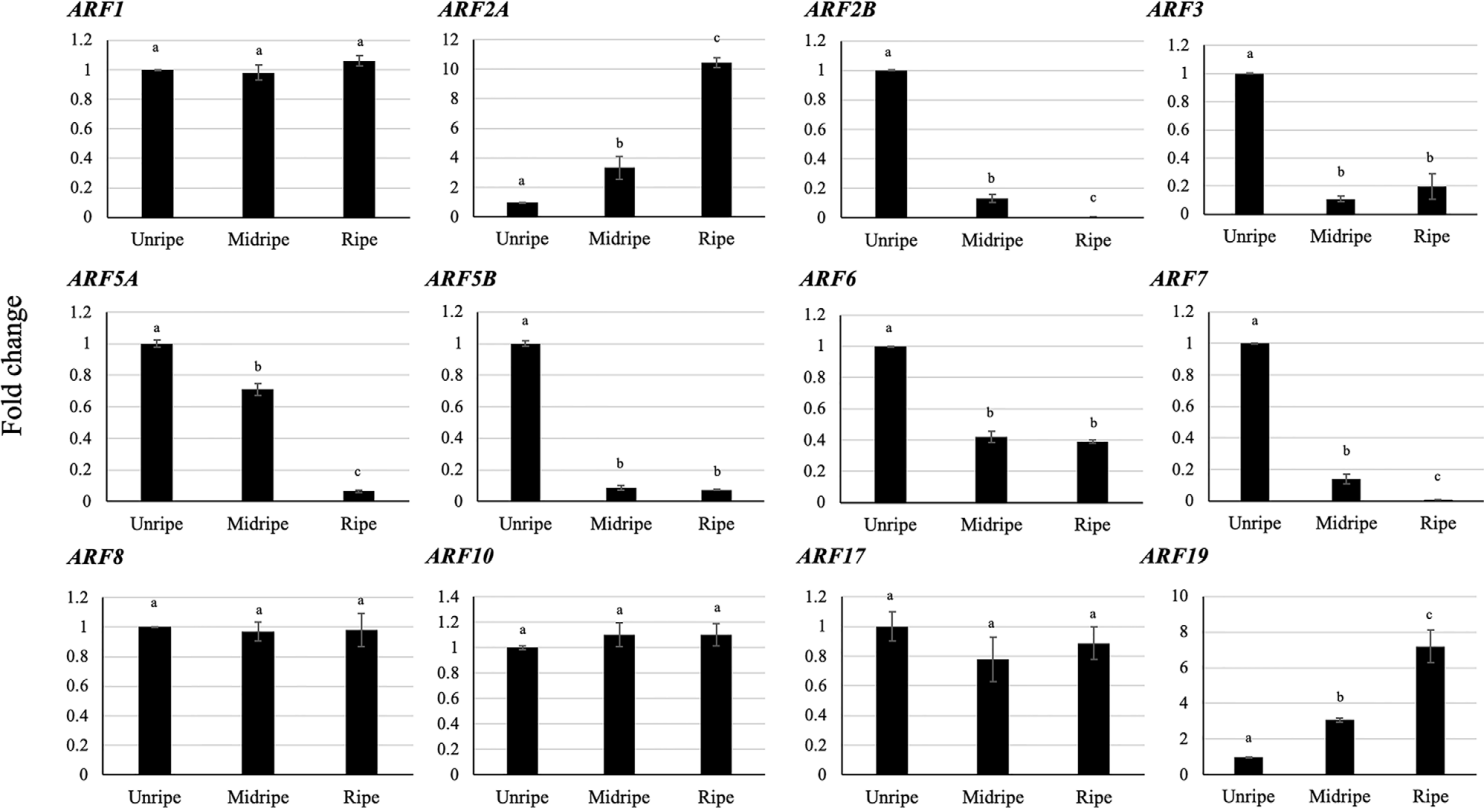
Fold changes in expression levels of *DzARF*s at three different stages (unripe, midripe, and ripe) during post-harvest ripening of durian fruit (Monthong cultivar). The relative gene expression levels were calculated by using the 2^-ΔΔCt^ method and levels were normalized by the geometric mean of reference genes and the unripe stage as control. Three independent biological replicates were used. Bars with different letters show significant differences (*P* < 0.05).

We used RT-qPCR to verify the expression patterns of *DzARF*s in three stages of the post-harvest ripening (unripe, midripe, and ripe) and identified 12 pulp-expressed *DzARF*s that were similar to those found in the Musang King cultivar. Notably, eight out of these 12 *DzARF*s (*DzARF2A*, *DzARF2B*, *DzARF3*, *DzARF5A*, *DzARF5B*, *DzARF6*, *DzARF7*, and *DzARF19*) harbored a ripening-associated expression pattern and were considered as putative ripening-associated TFs. The expression levels of the remaining four *DzARF*s (*DzARF1*, *DzARF8*, *DzARF10*, and *DzARF17*) did not vary significantly over the course of post-harvest ripening (**Figure 3**) and therefore these four *DzARF*s were not considered as ripening-associated TFs. Among the eight ripening-associated *DzARF* candidates, the *DzARF2A* and *DzARF19* transcripts were up-, while the *DzARF2B*, *DzARF3*, *DzARF5A*, *DzARF5B*, *DzARF6*, and *DzARF7* transcripts were down-regulated during post-harvest ripening. The expression levels of these eight putative ripening-associated *DzARF*s were further investigated under ethylene treatment.

### Validating Transcript Levels of Putative Ripening-Associated *DzARF*s Under Three Different Ripening Conditions

**Figure 4:**
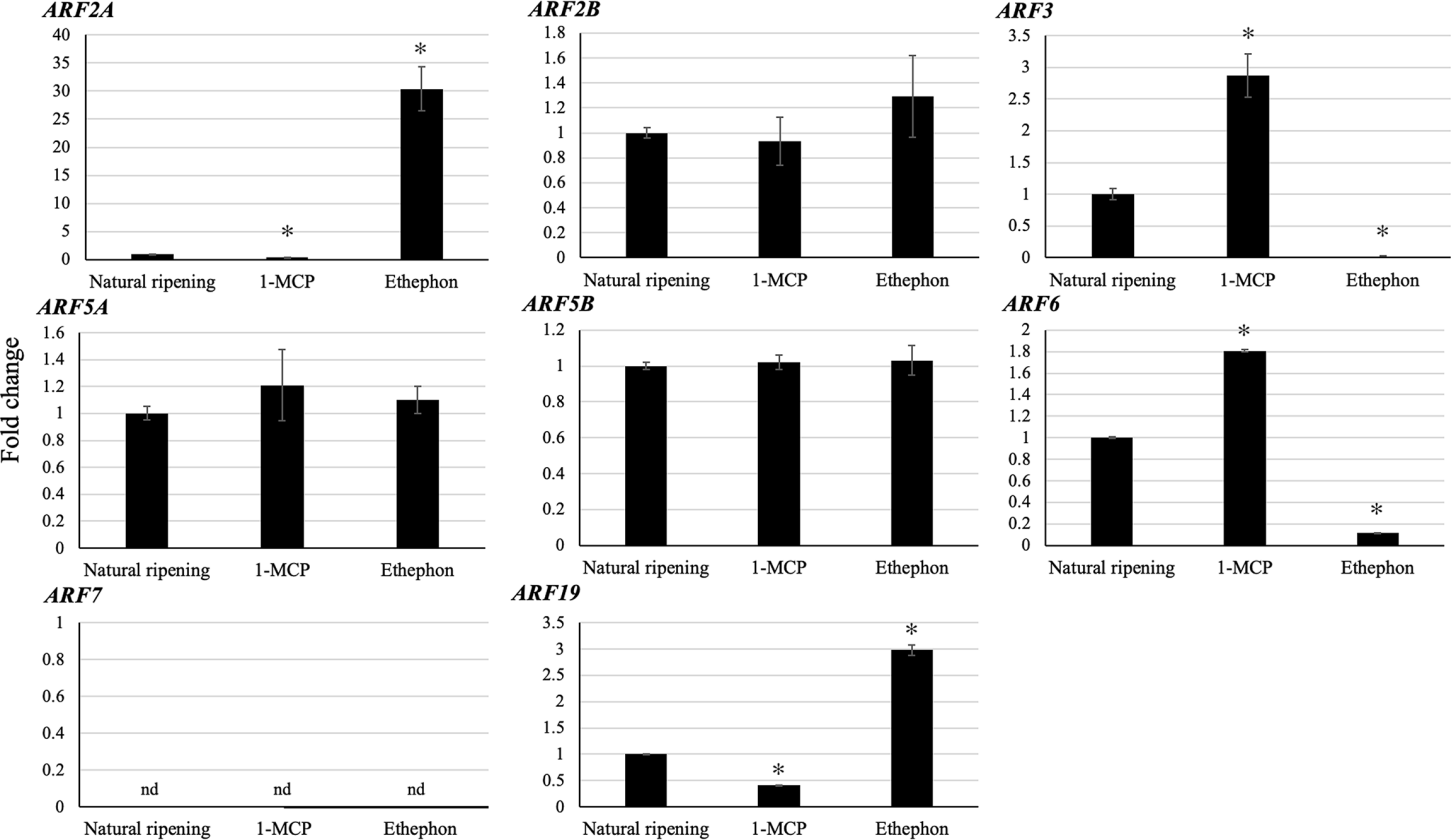
Fold changes in expression levels of eight candidate ripening-associated *DzARF*s under three different ripening conditions. The relative expression levels of candidate ripening-associated *DzARF*s were quantified under three different ripening conditions; natural (control), ethylene-induced, and 1-MCP-delayed ripening by using the 2^-ΔΔCt^ method and levels were normalized by the geometric mean of reference genes and the natural ripening condition as control. Three independent biological replicates were used. An asterisk (*) above the bar indicates a significant difference (*P* < 0.05) (nd = not detected).

Since ethylene plays a key role in climacteric fruit ripening, we analyzed the expression levels of the eight putative ripening-associated *DzARF*s under three different ripening conditions: ethephon-induced, natural, and 1-methylcyclopropane (1-MCP)-delayed ripening. Among these, the expression levels of *DzARF2A* and *DzARF19* were significantly induced under ethephon and suppressed under 1-MCP treatment, compared to those under natural ripening (control). However, the expression levels of *DzARF3* and *DzARF6* were significantly induced by 1-MCP and suppressed by ethephon relative to the control (**Figure 4**). Taken together, these results provide compelling evidence on the ripening-associated roles of these four DzARFs during post-harvest ripening in the durian fruit. Notably, the expression levels of *DzARF2B*, *DzARF5A*, and *DzARF5B* did not vary significantly under ethephon and 1-MCP treatments (compared to the control), so they were not considered as ripening-associated TFs. *DzARF7*, on the other hand, was not expressed under any examined condition, and thus it was also not considered as a ripening-associated TF. Among the validated ripening-associated *DzARF*s, *DzARF2A* displayed a dramatic increase of transcript accumulation under ethephon treatment and it was expressed increasingly during the post-harvest ripening of the durian fruit, suggesting a potential role as a transcriptional activator of ripening. This marked ripening-associated expression pattern of *DzARF2A* prompted its functional characterization to gain a better understanding of its physiological significance during fruit ripening in durian.

According to our phylogenetic analysis, DzARF2A was paired with ARF2 from tomato (SlARF2A and 2B) in subclade C3 (**Figure 1A**). Therefore, SlARF2A seemed to be DzARF2A’s closest tomato orthologue. Since down-regulation of *SlARF2A* resulted in ripening defects, decreased levels of climacteric ethylene production, and a decreased expression of the ethylene biosynthetic genes in tomato (Hao et al., 2015), it raised the possibility of a similar role by DzARF2A in the transcriptional regulation of ethylene biosynthetic genes during fruit ripening in durian.

### Transient Expression of *DzARF2A* in *Nicotiana benthamiana*

**Figure 5:**
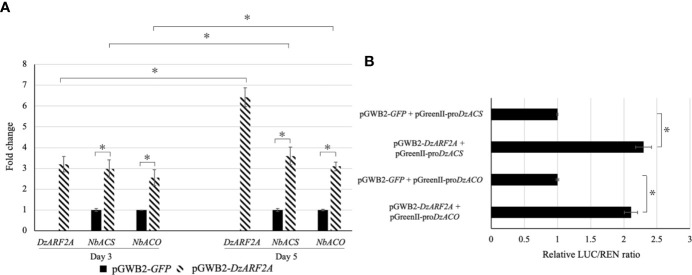
Fold changes in expression levels of *DzARF2A* and ethylene biosynthetic genes in *Nicotiana benthamiana* leaves transiently expressing *DzARF2A* and *in vivo* transcriptional activity of DzARF2A in *N*. *benthamiana* leaves. **(A)** The relative expression levels of *DzARF2A* and ethylene biosynthetic genes from *N. benthamiana*; ACC synthase (*NbACS*) and ACC oxidase (*NbACO*) were measured in *N. benthamiana* leaves infiltrated with pGWB2-*DzARF2A* (treatment) and pGWB2-*GFP* (control) at three and five days after infiltration by using the 2^-ΔΔCt^ method. The levels were then normalized by the geometric mean of reference genes and the pGWB2-*GFP* as control. Three independent biological replicates were used. An asterisk (*) above the bars indicates a significant difference (*P* < 0.05). In the *DzARF2A*-infiltrated leaves, comparisons were also made between day 3 and day 5. An asterisk (*) above the bars indicates a significant difference between day 3 and day 5 (*P* < 0.05). **(B)** After three days of infiltration, firefly luciferase (LUC) and *Renilla* luciferase (REN) activities were measured and presented as relative LUC/REN ratio. The LUC/REN ratio in the control leaves co-infiltrated with the pGWB2-*GFP* and pGreenII-pro*DzACS* or pGreenII-pro*DzACO* was set as 1 and was used as a calibrator. Error bars represent means ± standard deviations (SD) among four independent biological replicates. An asterisk (*) above the bars indicates a significant difference compared to the control (Student’s *t*-test, *P* < 0.05).

To gain insights into the *in planta* function of DzARF2A during the post-harvest ripening of durian, we transiently expressed it in *N. benthamiana* leaves. We found a significantly higher expression levels of ethylene biosynthetic genes (*NbACS* and *NbACO*) in the leaves infiltrated with *DzARF2A* compared to the control at day 3 and 5 post-infiltration (**Figure 5A**). In addition, our *in silico* analyses of the 2-kb promoter regions located upstream of the translation start site of *NbACS* and *NbACO* confirmed the existence of cis-regulatory binding sites (TGTCTC) for ARFs (**Supplementary Figure S3**). Notably, the 2-kb upstream region of the transcription start site should cover the putative promoter region of a gene (Jo and Choi, 2019; Liu et al., 2020). We compared the expression levels of *DzARF2A*, *NbACS*, and *NbACO* in the *DzARF2A*-infiltrated leaves between day 3 and 5 and found a strong positive correlation between the expression levels of *DzARF2A* and ethylene biosynthetic genes (**Figure 5A**). Taken together, our results strengthen the possible ripening-associated role of DzARF2A during durian fruit ripening through the transcriptional regulation of ethylene biosynthetic genes. To further confirm this role, the transcriptional activity of DzARF2A was investigated using dual-luciferase reporter assay.

### *In Vivo* Transcriptional Activity of DzARF2A

We performed dual-luciferase assay to evaluate the transcriptional activity of DzARF2A *in vivo*. Our effector plasmid contained the *DzARF2A* under the control of a CaMV35S promoter (pGWB2-*DzARF2A*) whereas our double-reporter vector harbored an internal control REN driven by the CaMV35S promoter and LUC driven by the 2000-bp promoter region of *ACS* (pGreenII-pro*DzACS*) or *ACO* (pGreenII-pro*DzACO*) (**Supplementary Figure S4**). The relative LUC/REN ratios in the leaves co-expressing both effector (pGWB2-*DzARF2A*) and reporter (pGreenII-pro*DzACS* or pGreenII-pro*DzACO*) constructs were compared to the control leaves co-infiltrated with pGWB2-*GFP* and reporter (pGreenII-pro*DzACS* or pGreenII-pro*DzACO*) plasmids. As shown in **Figure 5B**, compared to the control, the co-expression of DzARF2A with each of the two promoters significantly increased the relative LUC/REN ratio, suggesting that DzARF2A trans-activates ethylene biosynthetic genes.

### Expression Level of Candidate Ripening-Associated *DzARF2A* Among Different Cultivars

**Figure 6:**
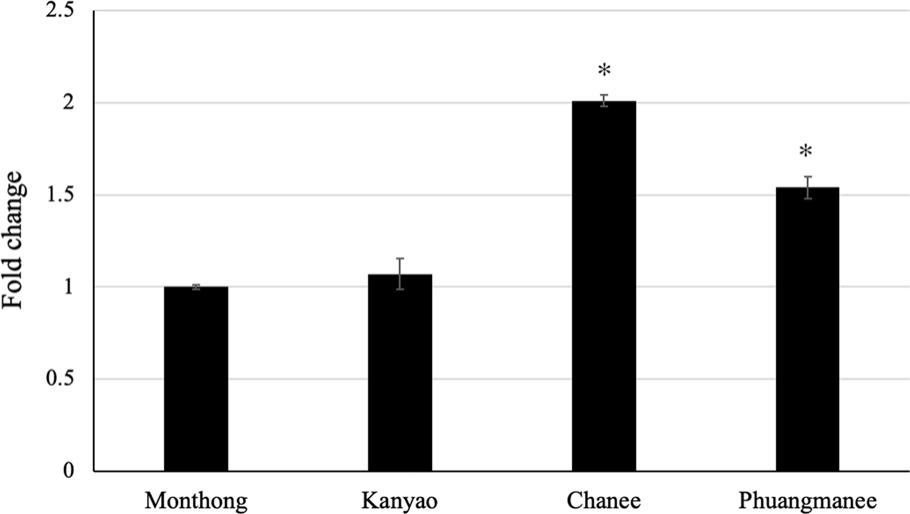
Fold changes in expression level of *DzARF2A* at ripe stage of different durian cultivars. The relative expression level of *DzARF2A* was analyzed in fruit pulps of two fast-ripening (Chanee and Phuangmanee) and two slow-ripening (Monthong and kanyao) cultivars at ripe stage by using the 2^-ΔΔCt^ method and levels were normalized by the geometric mean of reference genes and the Monthong cultivar as control. Three independent biological replicates were used. An asterisk (*) above the bar indicates a significant difference (*P* < 0.05).

It was our great interest to investigate any possible correlation between the expression level of candidate ripening-associated *DzARF2A* during ripening and the ripening behaviors of different durian cultivars. Therefore, we analyzed the expression level of *DzARF2A* in four cultivars; two fast-post-harvest ripening (Chanee and Phuangmanee) and two slow-post-harvest ripening (Monthong and Kanyao) cultivars. We observed a significantly higher expression level of *DzARF2A* in the fast-ripening cultivars compared to the slow-ripening ones (**Figure 6** and **Supplementary Figure S5**). This higher *DzARF2A* expression could therefore enhance the climacteric ethylene biosynthesis through the transcriptional regulation of ethylene biosynthetic genes and thus contribute to the faster ripening of Chanee and Phuangmanee, compared to the Monthong and Kanyao cultivars.

### Expression Level of *DzARF2A* Under Exogenous Auxin Treatment

**Figure 7:**
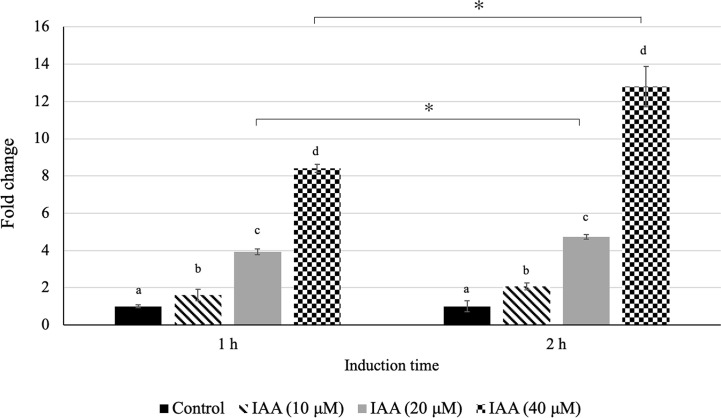
Auxin responsiveness of *DzARF2A*. Fold changes in expression level of *DzARF2A* in young leaves of Monthong cultivar treated with 0 (control), 10, 20, and 40 μM IAA for 1 and 2 h were calculated by using the 2^-ΔΔCt^ method and levels were normalized by the geometric mean of reference genes and the control samples (0 μM IAA). Three independent biological replicates were used for each condition at each timepoint. Different letters indicate significant differences (*P* < 0.05). Comparisons were also made for each condition between the two timepoints; an asterisk (*) above the bars indicates a significant difference for that condition between 1 and 2 h (*P* < 0.05).

In our previous study, we observed an increasing level of auxin during fruit ripening in both Monthong and Chanee cultivars with a significantly higher auxin level in Chanee compared to Monthong (Khaksar et al., 2019). This observation highlighted the possible ripening-associated role of auxin during durian fruit ripening and prompted us to further investigate whether there is a positive correlation between auxin levels and *DzARF2A* expression. Accordingly, we aimed to investigate the auxin inducibility of *DzARF2A* expression. Indeed, the accumulation of *DzARF2A* transcript was induced by exogenous auxin in the leaves of Monthong cultivar. We observed a significantly higher transcript abundance of *DzARF2A* in the leaves with increasing concentration of auxin and expression was significantly higher than that of the control (mock treatment) (**Figure 7**). Among the three auxin treatments (10, 20, and 40 µMu), exogenous auxin treatment at 40 µM elicited the highest expression of *DzARF2A* (**Figure 7**). Notably, auxin-induced expression of *DzARF2A* also depended on induction time and the highest expression level occurred at 40 µM after 2 h auxin exposure (**Figure 7**). This finding confirmed the auxin responsiveness of *DzARF2A* in a dose- and time-dependent manner. Moreover, it strengthened the possibility that DzARF2A could mediate the post-harvest ripening of durian fruit in harmony with auxin.

We found an increasing expression of *DzARF2A* in the leaves treated with different concentrations of exogenous auxin for 2 h. To further examine any correlation between the expression levels of *DzARF2A* and ethylene biosynthetic genes of durian, we profiled the expression levels of *DzACS* and *DzACO* in those leaves. The expression levels of *DzACS* and *DzACO* were positively correlated with increase in the expression level of *DzARF2A* (**Supplementary Figure S6**), consistent with our observation in *N*. *benthamiana* leaves (**Figure 5A**).

## Discussion

Fruit ripening is described as a well-orchestrated coordination of several steps, marked by major physiological and biochemical changes, and regulated by a complex hormonal network. Ethylene is regarded as the major regulator of climacteric fruit ripening. However, without minimizing the role of ethylene, the putative role of other phytohormones in regulating the climacteric ripening process remains elusive. Auxin, the most ubiquitous hormone in plants, is a crucial phytohormone involved in the regulation of various growth and developmental processes. In recent years, an increasing body of evidence has pointed towards a possible ripening-associated role of auxin in climacteric fruit ripening, with a number of emerging studies in peach, tomato, and durian. Auxin signaling is known to regulate the expression of early/primary auxin response genes through ARFs. Previous studies have already identified members of the ARF family in crops with economic values (Wang et al., 2007; Kumar et al., 2011; Xing et al., 2011; Luo et al., 2014; Wan et al., 2014; Shen et al., 2015; Hu et al., 2015; Liu et al., 2015; Singh et al., 2017; Tang et al., 2018). According to the Plant Transcription Factor Database (http://planttfdb.cbi.pku.edu.cn), 4,578 members of the ARF gene family exist in the genomes of different plant species in varying numbers; papaya (10), tomato (22), Arabidopsis (37), banana (50), and durian (77). Even though genetic and molecular studies have begun documenting the important roles of ARF gene family members in several aspects of plant growth and development, such as AtARF1, AtARF2, AtARF7, and AtARF8 in Arabidopsis and SlARF3 of tomato, the actual knowledge on the role of ARFs in fruit ripening remains strikingly limited. Durian is a typical climacteric fruit with restricted shelf life after the initiation of ripening. This limits the handling and transportation of the fruit, which can cause significant economic losses to both farmers and consumers. Therefore, a deeper understanding of the molecular mechanisms behind ripening and the different ripening behaviors of durian cultivars (e.g., fast and slow post-harvest ripening) is critical to improve their post-harvest life. Our transcriptome-wide analysis of durian (Musang King cultivar) identified 15 non-redundant members of the ARF gene family. Out of these, our gene expression analysis identified 12 fruit pulp-expressed *DzARF*s during the post-harvest ripening of the Monthong cultivar, from which eight *ARF*s harbored ripening-associated expression patterns (**Figure 3**). The expression levels of these putative ripening-associated *DzARF*s were further examined under three different ripening conditions. The expression levels of *DzARF2A* and *DzARF19* were induced under ethephon and suppressed under 1-MCP (**Figure 4**). The expression of these ethylene-induced *DzARF*s showed an increasing trend during the post-harvest ripening of durian and are speculated to act as transcriptional activators of ripening. Similar to our observation, Hao et al. (2015) identified a member of the ARF gene family in tomato (SlARF2A) which was ethylene-induced and acted as a transcriptional activator of ripening. In contrast, the expression levels of *DzARF3* and *DzARF6* were significantly suppressed under ethylene but induced under 1-MCP and showed a decreasing trend during ripening. According to our knowledge, this is the first report on the inducing effect of 1-MCP on the expression of an ARF gene. Since the inhibition of ethylene biosynthesis (Kieber et al., 1993; Tatsuki and Endo, 2006) and signaling (Hall et al., 2000) by 1-MCP is well documented, a plausible explanation for the upregulation of *DzARF3* and *DzARF6* by 1-MCP through the inhibition of ethylene biosynthesis and signaling is possible since the expression of these two genes was negatively regulated by ethylene. Thus, these two ethylene-repressed *DzARF*s possibly act as transcriptional repressors of ripening. A similar observation was made in our previous study, where we identified both ethylene-induced and ethylene-repressed Dof TFs in durian (Khaksar et al., 2019).

From the validated ripening-associated and ethylene-induced *DzARF*s, the dramatic increase in the expression level of *DzARF2A* under ethylene and its significantly higher expression level during fruit ripening, compared to that of *DzARF19* (**Figure 2**), motivated us to characterize it further. Transient expression of *DzARF2A* in *N*. *benthamiana* leaves significantly upregulated the expression levels of the ethylene biosynthetic genes (*NbACS* and *NbACO*) (**Figure 5A**), confirming the transcriptional regulation of these genes by DzARF2A. This observation is in line with the results of previous studies on *ARF2*. Specifically, the expression of *ACS* genes was downregulated in the Arabidopsis *arf2* mutant (Okushima et al., 2005) and Hao et al. (2015) showed that tomato fruits under-expressing *SlARF2A/B* produced less climacteric ethylene and exhibited a dramatic down-regulation of the ethylene biosynthetic genes. Our *in silico* analyses of the *ACS* and *ACO* promoter regions from durian and *N*. *benthamiana* revealed the existence of cis-regulatory binding sites (TGTCTC) that are needed to interact with ARFs (**Supplementary Figures S7** and **S3**). Using dual-luciferase reporter assay, we then confirmed the transcriptional activation ability of DzARF2A (**Figure 5B**). Taken together, these observations strengthen the possible role of DzARF2A in the regulation of fruit ripening through the transcriptional regulation of ethylene biosynthetic genes in durian.

In our previous study, we profiled the expression levels of ethylene biosynthetic genes (*DzACS* and *DzACO*) during durian fruit ripening of Monthong and Chanee cultivars. Both genes were found to express increasingly over the course of ripening in both cultivars (Khaksar et al., 2019). Notably, the increasing expression levels of *DzACS* and *DzACO* were consistent with the expression level of *DzARF2A* during ripening. In addition, we observed significantly higher expression levels of *DzACS* and *DzACO* during post-harvest ripening in Chanee (a fast-ripening cultivar), compared to Monthong (a slow-ripening cultivar) (Khaksar et al., 2019). This observation prompted us to further examine any possible correlation between the expression levels of *DzARF2A* and ethylene biosynthetic genes during durian fruit ripening. We observed a strong positive correlation between the expression levels of *DzARF2A* and ethylene biosynthetic genes (**Figure 5A** and **Supplementary Figure S6**). Moreover, the expression level of *DzARF2A* was positively correlated with the faster post-harvest ripening of durian fruit (**Figure 6**). Thus, higher *DzARF2A* expression would upregulate climacteric ethylene biosynthesis and lead to a faster ripening of the fast-ripening cultivars (Chanee and Phuangmanee) compared to those of the slow-ripening ones (Monthong and Kanyao). To the best of our knowledge, this is the first report on the role of a ripening-associated TF that positively regulates the post-harvest ripening of a climacteric fruit in a cultivar-dependent manner; in other words, the higher the *DzARF2A* expression (in fast-ripening cultivars), the faster the ripening. It has been postulated that the regulatory network mediating fast- and slow-ripening cultivars would involve not only DzARF2A and ethylene as master regulators, but also other TFs and hormones. We observed an exogenous auxin-induced expression pattern of *DzARF2A* in a concentration-dependent manner (**Figure 7**), which was similar to that of *SlARF2A* in tomato (Breitel et al., 2016) and *CpARF2* of papaya (Liu et al., 2015), suggesting that *ARF2* has an evolutionarily conserved function in regulating fruit ripening. Thus, the auxin responsiveness of *ARF2* in these climacteric fruits strengthened the active involvement of auxin during climacteric fruit ripening.

In our previous study, we observed an increasing level of auxin during ripening in both Monthong and Chanee cultivars and a significantly higher auxin level during post-harvest ripening in Chanee over Monthong. This phenomenon coincided with the significantly higher expression levels of auxin biosynthetic genes in Chanee compared to those of Monthong (Khaksar et al., 2019). In addition, we identified the cultivar-dependent DzDof2.2, a member of the Dof TF family, which was expressed at a significantly higher level during ripening in Chanee compared to Monthong. Functional characterization of *DzDof2.2* suggested its role in controlling fruit ripening through the transcriptional regulation of the auxin biosynthetic genes (Khaksar et al., 2019). Based on these previous findings and the results of our current study, DzARF2A might act jointly with DzDof2.2 in a regulatory network to control post-harvest ripening in durian. In the fast-ripening cultivars, higher *DzDof2.2* expression would enhance auxin biosynthesis. Higher auxin level would consequently upregulate the expression of *DzARF2A* and activate the ARF2A-mediated transcription of ethylene biosynthetic genes, leading to a higher climacteric ethylene biosynthesis (auxin-ethylene crosstalk) and faster ripening.

The present study documents for the first time a regulatory network that controls fast and slow post-harvest ripening in different cultivars of a climacteric fruit, in which DzARF2A interconnects signals from ethylene and auxin to fine-tune and to coordinate the initiation of ripening. The relationship between ethylene and auxin in plant growth, development, and fruit senescence has been previously documented (Iqbal et al., 2017). In addition, Bleecker and Kende (2000) pointed out that auxin can stimulate climacteric ethylene biosynthesis through its inductive action on the expression of ethylene biosynthetic genes. Besides the already known role of auxin to induce climacteric ethylene synthesis and thereby to influence ripening, the assumption that auxin would play a direct role during climacteric ripening, even though previously formulated, due to the lack of clear experimental evidence is still somewhat uncertain. A study by El-Sharkawy et al. (2016) suggested a possibly direct role of auxin in plum fruit ripening through the upregulation of several genes encoding cell-wall metabolism-related proteins. However, the intriguing question about which ripening-associated genes could be auxin responsive remains open and needs further investigation.

Often, different TFs control the expression of a particular gene and the fine-tuning of these different TFs occurs through the formation of enhanceosome or repressosome complexes that affect protein–protein and protein–DNA interactions (Martinez and Rao, 2012). Various ripening-associated TFs have been investigated. However, only a few studies have indicated their involvement in fruit ripening, such as the studies in tomato (Shima et al., 2013; Fujisawa et al., 2014) and in banana (Feng et al., 2016). To the best of our knowledge, there has only been one study that reported a possible ARF interactor in the regulation of ripening. Breitel et al. (2016) identified the ASR1 (ABA STRESS RIPENING-INDUCED 1) protein as a putative ARF2A interactor in tomato fruit. They suggested the possibility that these two proteins could be coordinating the interaction between ethylene and ABA during tomato ripening. The possible interaction between DzARF2A and other TFs, such as DzDof2.2 in controlling fruit ripening in durian could be the subject of a further investigation.

In summary, out of the 15 identified *DzARF*s, 12 were expressed in the fruit pulp. Gene expression analysis revealed differential expression of the fruit pulp-expressed *DzARF*s during post-harvest ripening in the Monthong cultivar. A total of eight *DzARF*s harbored a ripening-associated expression pattern, suggesting a potential role in fruit ripening. Among those, the appealing ripening-associated expression pattern of *DzARF2A* prompted its further functional characterization and confirmed its role in ripening through binding to the promoters of ethylene biosynthetic genes and transcriptionally regulating their expression levels. We observed a positive correlation between the expression level of *DzARF2A* and ripening speed in the fast-ripening cultivars (Chanee and Phuangmanee). Taken together, our results propose DzARF2A as a component of the regulatory network underlying fruit ripening in durian. Thus, the present study on durian suggests another layer in the complex regulatory networks behind fruit ripening and it strengthens the concept that the ripening process relies on the interplay between different transcription factors and hormones. Further functional analysis of DzARF2A in fruits would provide more insights into its ripening-associated role during durian fruit ripening.

## Data Availability Statement

The datasets presented in this study can be found in online repositories. Illumina sequencing reads from RNA-Seq study of durian (Musang King cultivar) were retrieved from a public repository database (SRA, Sequence Read Archive) with the following accession numbers: SRX3188225 (root tissue), SRX3188222 (stem tissue), SRX3188226 (leaf tissue), and SRX3188223 (aril/pulp tissue).

## Author Contributions

SS conceived the research. GK performed the experiments. GK and SS designed the experiments, analyzed the data, and wrote the manuscript. All authors contributed to the article and approved the submitted version.

## Funding

This research is supported by the Thailand Research Fund (RSA6080021) and Chulalongkorn University (GRU 6203023003-1) (to SS).

## Conflict of Interest

The authors declare that the research was conducted in the absence of any commercial or financial relationships that could be construed as a potential conflict of interest.
