# Supplementary material for: Auxin Response Factor 2A Is Part of the Regulatory Network Mediating Fruit Ripening Through Auxin-Ethylene Crosstalk in Durian
Source: Front Plant Sci. 2020 Sep 9;11:543747. doi: 10.3389/fpls.2020.543747 (PMC7509138; doi:10.3389/fpls.2020.543747)
Supplement: Supplementary file 1 [file DataSheet_1.docx]

Supplementary Material

Supplementary Figures and Tables

**Supplementary Table S1. List of primers for *DzARF*s and reference genes used in this study**

| Gene name | Forward primer (5'-3') | Reverse primer (5'-3') |
| --- | --- | --- |
| *ARF1* | TCTCCTCCAGGTGCTCAAATG | CACATCCAAATCAGCAGGGT |
| *ARF2A* | GGACATGCTGTGGAGGTAGTAG | TCTCTGTACAAGGAGGGTGGT |
| *ARF2B* | ATGCAGCCATGAACTTTCTCC | AAGCTAGCCTGACTAAACAAACC |
| *ARF3* | CAAACCCCATGCAGTTCCATTC | GGGACGATCCCGTCATATTCG |
| *ARF4* | GCCTTTCTGGCCTTATGGTATT | CAACGCATGAATCAGTTTTGGAGA |
| *ARF5A* | GCAATTCGGGTGCGATCTTC | TGCTAGAAGTTCATTTCCTGCTG |
| *ARF5B* | ATCCTTGGGACGAATTTGTTGG | ATTGCAACCTGAATCGAAGCC |
| *ARF6* | TGTCCGGACTCTTAAGCACC | CAGTTGTGTTTAAGCCAGCCC |
| *ARF7* | TCAACAGATCCCGAGGCTGAT | TTGGCACCGACACCTGAGTT |
| *ARF8* | CAGTAGCCCGACATTGTGTGT | CCTGGGCTGTGAACTTGATAC |
| *ARF9* | GTTACTTTGGGCTCTGGAGGA | TCTTCAAACCCAGGTGATGTCT |
| *ARF10* | TCAGTTTTATGTTGATATTGACCGC | AATTGCATTTTGAAGCATATGTGGA |
| *ARF17* | TCAAACTCTGGATGATTGGAGCA | CCAAGCGTATTTATACCAGTGCC |
| *ARF18* | GGGGATGTCTGCATAGGTTTCT | GCATGGATGCATGGAGTCATGATAA |
| *ARF19* | CTGCATCGATAGTTTGGAAGGG | TAGTCGAAACCAAGCCATACA |
| ^*^*DzEF-1α* | GAAACCTTCTCTGCGTACC | CTCCACACTCTTGATGACAC |
| ^**^*DzACT* | AATGAGCAAAAAGGGTCAGCAC | GTCTTCAAAGTCAGCAGCCAG |

* Durian elongation factor 1 alpha (first reference gene)

** Durian actin (second reference gene)

**Supplementary Table S2. List of primers used for transient expression of *DzARF2A* in *Nicotiana benthamiana*, expression analysis of ethylene biosynthetic genes in *N. benthamiana* and durian leaves, and dual-luciferase reporter assay**

| Gene | Forward primer (5'-3') | Reverse primer (5'-3') |
| --- | --- | --- |
| For gene cloning  *DzARF2A* | AAAAAGCAGGCTATGACAACTACTTCGGAGGTATC | AGAAAGCTGGGTCTAAGAATTTTCTGCACTACATGCTG |
| For expression analysis of ethylene biosynthetic genes in *N*. *benthamiana* |  |  |
| *NbACS* | TCTCGAAAAGAATGTACGATGAAAG | ATTCCTCCGTTTTAAGTTCTAGCTC |
| *NbACO* | TGCATAGATCCAAATTCCAGACTAC | GTAGTAGGGACACACACTTTTGTA |
| ^*^*NbEF-1α* | TGAGGGACATGCGTCAAACT | GGAGCGGATACCAGTCATACA |
| ^**^*NbACT* | AGTGGCGGTTCGACTATGTTTC | TGCCTTTGCAATCCACATCTGTT |
| For expression analysis of ethylene biosynthetic genes in durian |  |  |
| *DzACS* | CAGAAATCATTGGCCTGGTAG | CTGAGTTGGAGCTGAAATGG |
| *DzACO* | TGGAGAAAGAAGCAGAGGAG | AAGTTGTTTCCCTGGCTTTC |
| For dual-luciferase reporter assay |  |  |
| Promoter amplification (*DzACS*) | CTTCGTAGTTCATCGTATTCATCTT | GAGAAGCAGAGAGTAGAGACGTG |
| Promoter amplification (*DzACO*) | TTCCAGGCCAAGGAACCAAG | ACATTCTCATTATAACTGCTCCACT |

Underlined sequences represent bases of the *att*B1 or *att*B2 site on the 5' end of each primer (for BP reaction)

* *Nicotiana benthamiana* elongation factor 1 alpha (first reference gene)

** *Nicotiana benthamiana* actin (second reference gene)

**
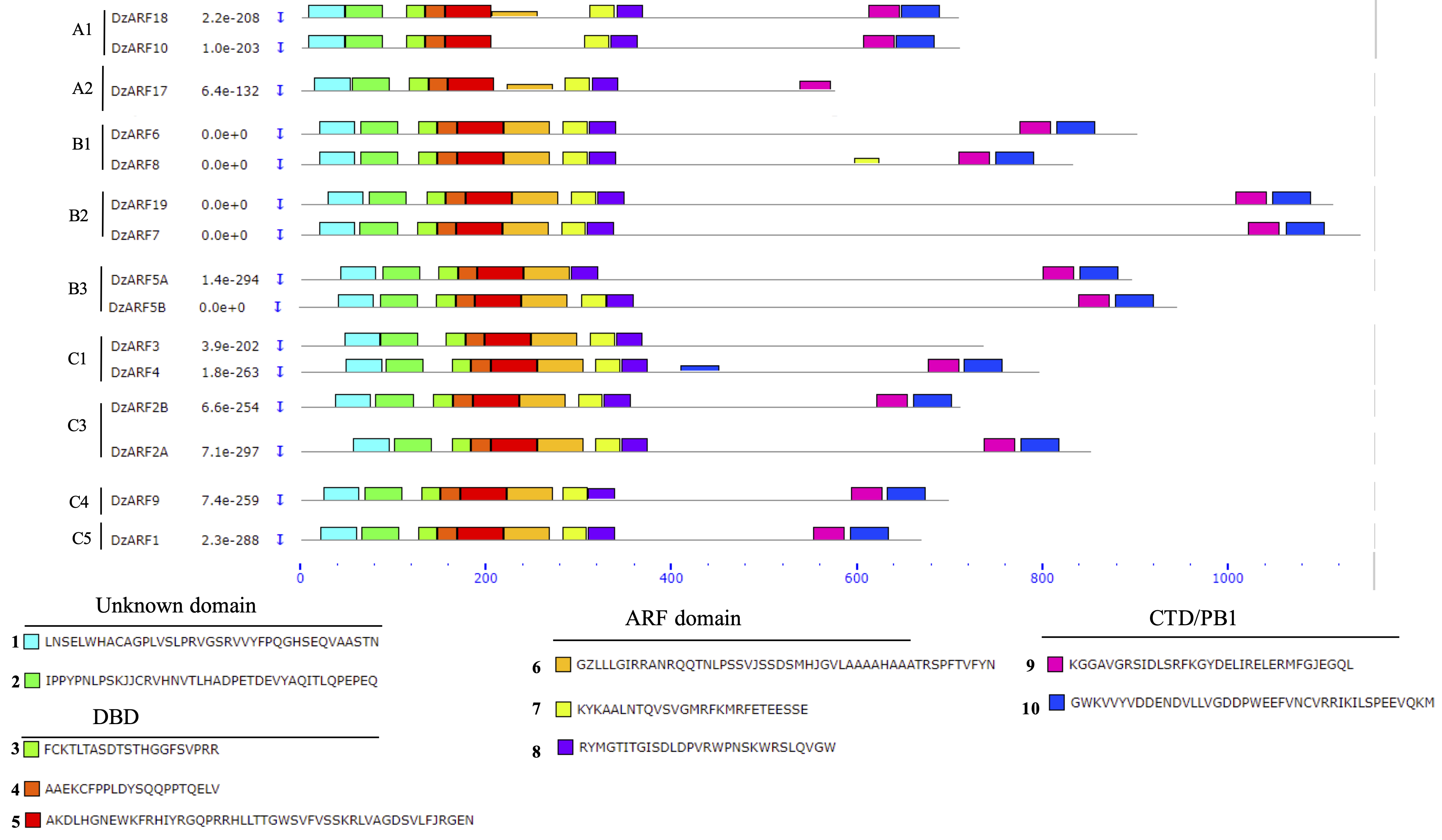
**

**Supplementary Figure S1** Motif organization of 15 DzARFs. Schematic distribution of ten conserved motifs identified by MEME suite version 5.1.0. is represented. Numbers indicate the motif numbers.

**
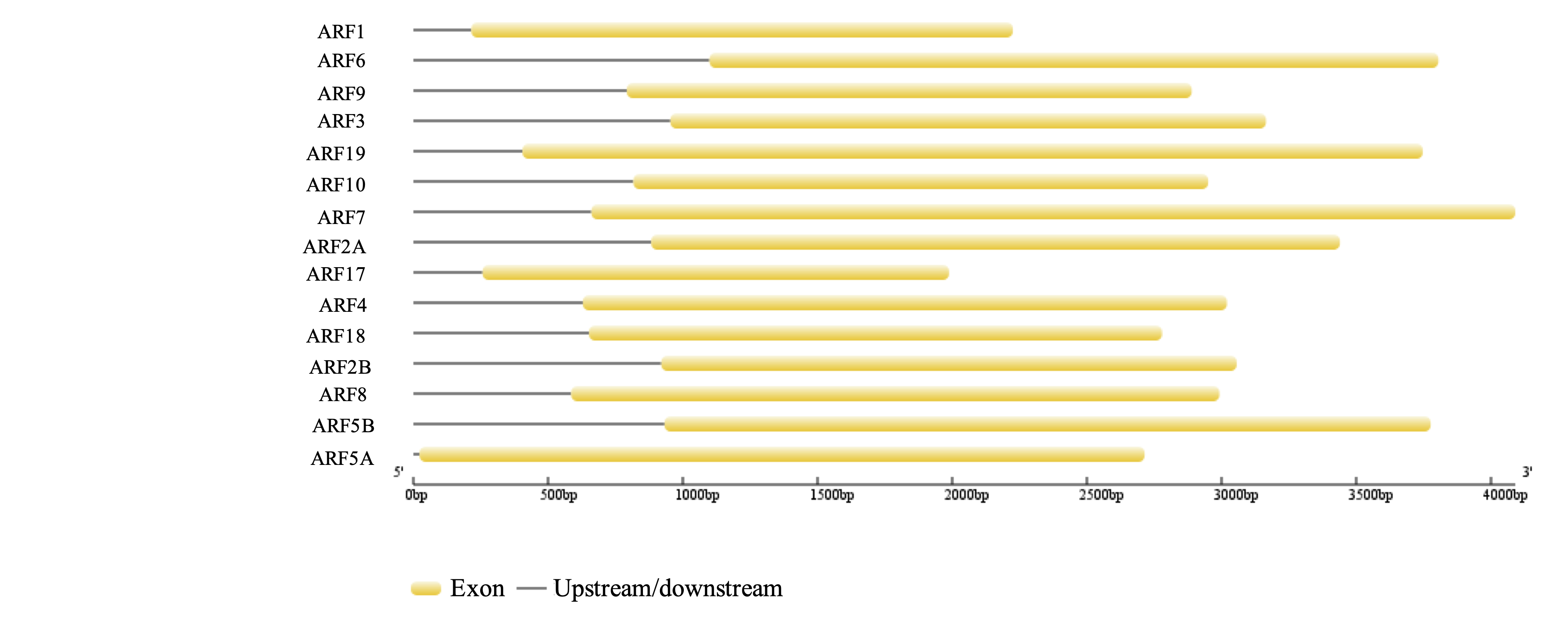
**

**Supplementary Figure S2** Gene structure of *DzARF*s. Exon-intron structures of *DzARF*s were analyzed using Gene Structure Display Server 2.0 (with default parameters).

***Nicotiana benthamiana ACC synthase* (*NbACS*)**

TTTTATTGCCATCTAATATTCTCCTACGTTATTTGGTGTTTCGCTACTTTAGTCCTTCTCCCTATATATA

TCCCTCACTTATTCCTCATTCTCTTCCCAACACCACTACAATCTAAGCAACCAGCTTTTCTATAGACAGA

AGTAGTAGTCACATATATATATATATATATTTTGATCCCATTTTCATCTTCTCCAATATTTTTTTCACTT

TAAACTCGTTAAGAAAAATGGGATTTGAGAATGAGAAGAACAGCTCAATCTTATCTAAGCTCGCTACTAA

TGAAGAACATGGTGAAAACTCGCCATATTTTGATGGATGGAAAGCATACGATAACGATCCTTTTCACCCT

CTGAAGAACCCTAATGGTGTTATCCAAATGGGTCTTGCTGAAAATCAGGTTTTTACTCAAACTTATGATT

CAGTATTCCTCTATGATTTCTATGTTCTTTACTATATTTGCTTTCTCTGAATTAAGAAGAAGATGATGCA

TGCTTATATATATATATATATATATTTTTTTTTTTTTTCTCACCTCGGTTTGTCTCATTTATTTGCAGCT

TTGTTTTGACTTGATTGAGGAATGGATTAAGAGAAATCCAAACGCTTCAATTTGCACCACAGAAGGAATC

AAATCTTTCAGGGCCATTGCCAACTTTCAAGATTATCACGGCCTACCTGAATTCAGAAGCGTACGTATAG

TATCACGCTATAGTCAGTTAAATTGCACTGATAATGTAAAACCTCGTCAGAATAATATGAGTTTAATTAT

ATTATATCTGTTGCAGGCTATTGCGAAATTTATGGAGAAAACAAGAGGGGGAAGGGTTACGTTTGATCCA

GAGAGAGTAGTTATGGCTGGTGGTGCAACTGGAGCCAACGAAACAATTATATTTTGTTTGGCTGATACAG

GCGATGCATTCTTAGTACCTTCACCATACTATCCAGCGTATGCATTTTTATTTTACTCATTATATTTTCA

TTTGTTTTATTTCTTGCATTCTCCAGGGAGTATATTTCGTTTTCCATATCTTTTTATGTTGACTATTACA

TTAAAGGAAATTAAAAGAAACAAGAAATAATGTGGGAATTTATTGTGTCTCTTTATGTTGGACGGCGTAA

CCATTGACTTGGAGAATTTAACCTTAATTGTCTCGGTCATCCACGTAAGCTCTTCAATTTTTCGTAATAT

TTTCCTGTAGTGGAATCCAGTTTGATGGTGAAGTTGGATAAACAGCTCAGTATTCACACTATATTATATT

TGATTAGGGAAAATAAGAGAAATGGAATTAGTGATATAACAATTGTCAAACAGACAAGTAAACCCAAAAT

GAAAAAGATGACTAAGGTATGTAGTGGTCAATTCTTCTGAACTTTTTAGAGTAATTGAGTACGCTCTTTC

CTGCTTGACCATGACACTCGAGCTAATATAAATTTTTAGACATTGATAATGACGTGTCAAAGATTTAAAA

GATACAAGTCAAGATTGCTGCTCCATTTGTATATATTCTCTTGTTTTCACTAAATGAACAATTTAACAAT

TTAAAGAAAAACAATAAAATAATAACTTGAAAGACCCACGTGGGGATGTTACAATACTTTTCTTTCTCTT

TTTCAATTTGGTCAATTGTTCTATATATAATACTCTAACTAATAATATTTTGGCAATTAATTAATTAATG

CAGATTTAACCGGGACTTAAGATGGAGAACTGGAGTACAACTCATTCCAATTCCTTGCGACAGTTCCAAC

AACTTCCAAATCACTACAAAAGCTGTGAGAGAAGCATATGAAAATGCCCAGAAATCAAACATCAAAGTCA

AAGGCTTGATTTTGACCAACCCATCAAATCCATTAGGCACCACTTTGGACAGAGACACACTGAAAAATCT

CTTGACCTTCACCAACCAACATAACATCCACCTCGTTTGCGACGAAATTTACGCCGCAACTGTCTCTAAT

ACACCTCAATTCGTCAGCATTGCTGAAATTCTCGACGACGATG

***Nicotiana benthamiana ACC oxidase* (*NbACO*)**

ATCCGCCGGTTAAATCGTTTTTTATCCATATTAAATATGGGTTGGGTCGGATAATTTATCCGTTTTTTAT

TATCCGTTTTCAATATGTCCCATAACCGACTCGACCTTCCCGTTTGCCACCCCTAGTCAGGTGAGTGATT

TTCCACATGGGAGACGTGGGATTGATTCTCCTCACCAACAGCCTCCTCAGTCAGAGTTCGTCGCACCTGA

CTTCCCTAGTGCGGTTTATATCTTCTGTATGGTTTGCGAACTATTGTACAGTGAACAGGAATTATCCAAT

GCGCAACCGAATGATAGCGGCTACGTGTTTCCCTGAGAATTTAAAAAAAAAAAAGAGTTACCCGATACAT

GTGCTGGTTGATAACAAGTATCACATGGACTAGTCGAGATGTGTGCAGATTCGGATATATATATATATAT

GTATATTATATATACTGTTATTAGAAGAAAAAACAAGAACTTCTAAACCTCAAGGATAATGAAGTATTAT

TTTAACATAAATTTTTTGGTTATTGGTTGTTATTATTATAATTATTATTATAATAAAGTAACATGATATA

AGTCAGTGTCCAACTTTGCCAGCCTAAATTGTCCAAGATTCCAAATTGGATACAGAAGAAGGACAAGGAT

TTACTATAACTGAACATGCTGCACCTGCTACCCCTTATTTTATTCTATTTTGTTTTCTTTTGTGTGTATG

TGGGGAAATGAGGGGTTGCATATGTACTTCAAAGATAAGTTTCTTCTTTAAAAAAATAACATATTGTTAA

TCCAAAGTTGTTCATATAATGCAGGGAGGTTATGAGAGATTTTGCTAAAAGATTAGAGAATCTAGCAGAG

GAGCTGTTGTATTTGCTCTGTGAAAATCTTGGTCTAGAAAAGGGATACTTGAAAAATGTATTTTATGGAT

CTAAAGGTCCAAACTTTGGAACTAAAGTGAGCAATTATCCACCATGCCCAAAACCAGATTTGATTAAGGG

ACTGCGCGCCCATACGGACGCTGGTGGCATAATCCTTCTCTTCCAAGATGACAAAGTAAGCGGCCTACAA

CTCCTCAAAGACGGCCAATGGATCGATGTTCCTCCTATGCGCCACTCCATCGTCGTTAACCTTGGCGATC

AACTTGAGGTAATCATCCAAAAATGTCTCAGTTAGGAGTTTGAATGAAGAGTTTGATGAGTCTGATGTAG

TAGATGCACGTGCACCAATTGATTATATGTTGGATCGAGCTAGGTGTCTCTAATGTCTCATTGGCGAGGC

GTCTAGTGTTTTTTCCCTTAAATTTTCTATATACATATACAAAGTTTGAGTCTTATGCAATAGGTGCACG

TGCAACAGGTCCACGTGCACCAACTGCTTATATGTTGACTCGAGCAACGTGTATACTTTTCTCACTATAG

ATTGGAGTATATACCAAGTGTTCTAATGAAAAATGTGAAAACAGGTGATCACAAATGGGAAGTACAAGAG

TGTGATGCACAGAGTGGTAGCGCAAAAAGACGGGACTCGGATGTCATTAGCTTCTTTCTATAATCCAGGA

AGTGATGCAGTGATTTATCCAGCACCAGCTCTTGTTGAGAAAGAGGCAGCGGAGAGCAAACAAGTTTATC

CCAAATTTGTGTTTGATGATTACATGAAGTTATACGCCGGACTAAAGTTTCAGGCCAAGGAGCCAAGGTT

TGAAGCCATGAAATCTATTGAATCTGATGTCAAGATGGATCCAATTGTAACTGCATAGATCCAAATTCAA

GACTACTAAAGATGAAAATTGAAAGAAAATGATTTGTTATTGAAGTAGTACAAGACGAGTGACATACATT

ATTTGCTTGTTTTGGTATAGTGGAATTAATATATTTACAAAAGTGTGTGTCCCTACTACATATGTAGTCA

AGAGGCTTAAAGTTTGTATCTTATAATAAACTGATAAAGTTTCCTTATACAGATTTTAATGTACCAAAAA

TTACCTGTGTTACTTCTTTTCTCATTGGATTTTGCCGTCAATG

**Supplementary Figure S3** Nucleotide sequences of promoter regions of ethylene biosynthetic genes from *Nicotiana benthamiana* (1-aminocyclopropane-carboxylase (ACC) synthase (*NbACS*) and ACC oxidase (*NbACO*)). ARF binding sites (TGTCTC) are highlighted in yellow. The translational start site (ATG) is underlined.

**
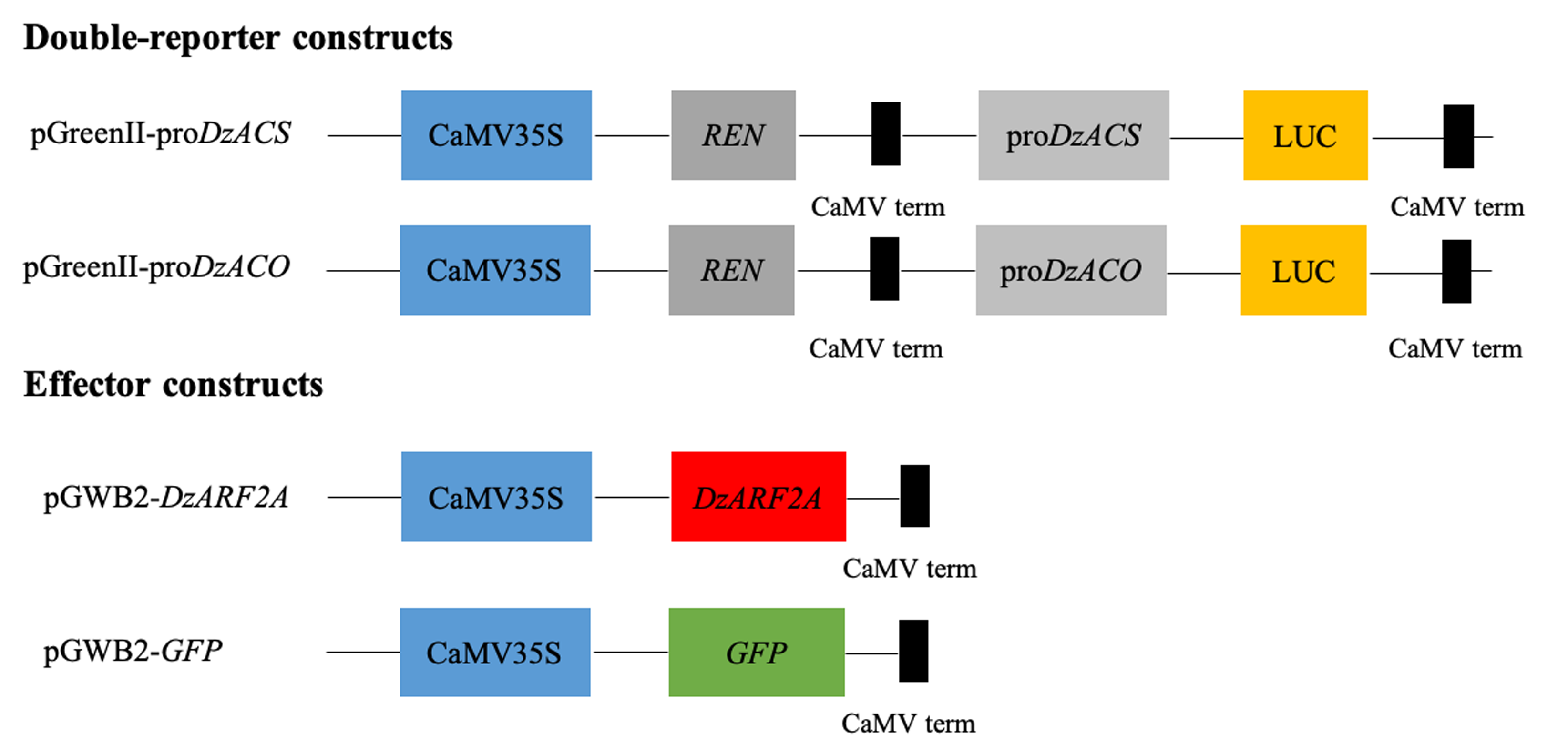
**

**Supplementary Figure S4** Schematic diagram of different constructs used for dual-luciferase reporter assay. The effector construct contained the DzARF2A driven by a CaMV35S promoter (pGWB2-DzARF2A). The pGreenII 0800-LUC double-reporter vector harboured an internal control REN under the control of the CaMV35S promoter and LUC driven by the 2000-bp promoter region of *DzACS* (pGreenII-pro*DzACS*) or *DzACO* (pGreenII-pro*DzACO*).

**
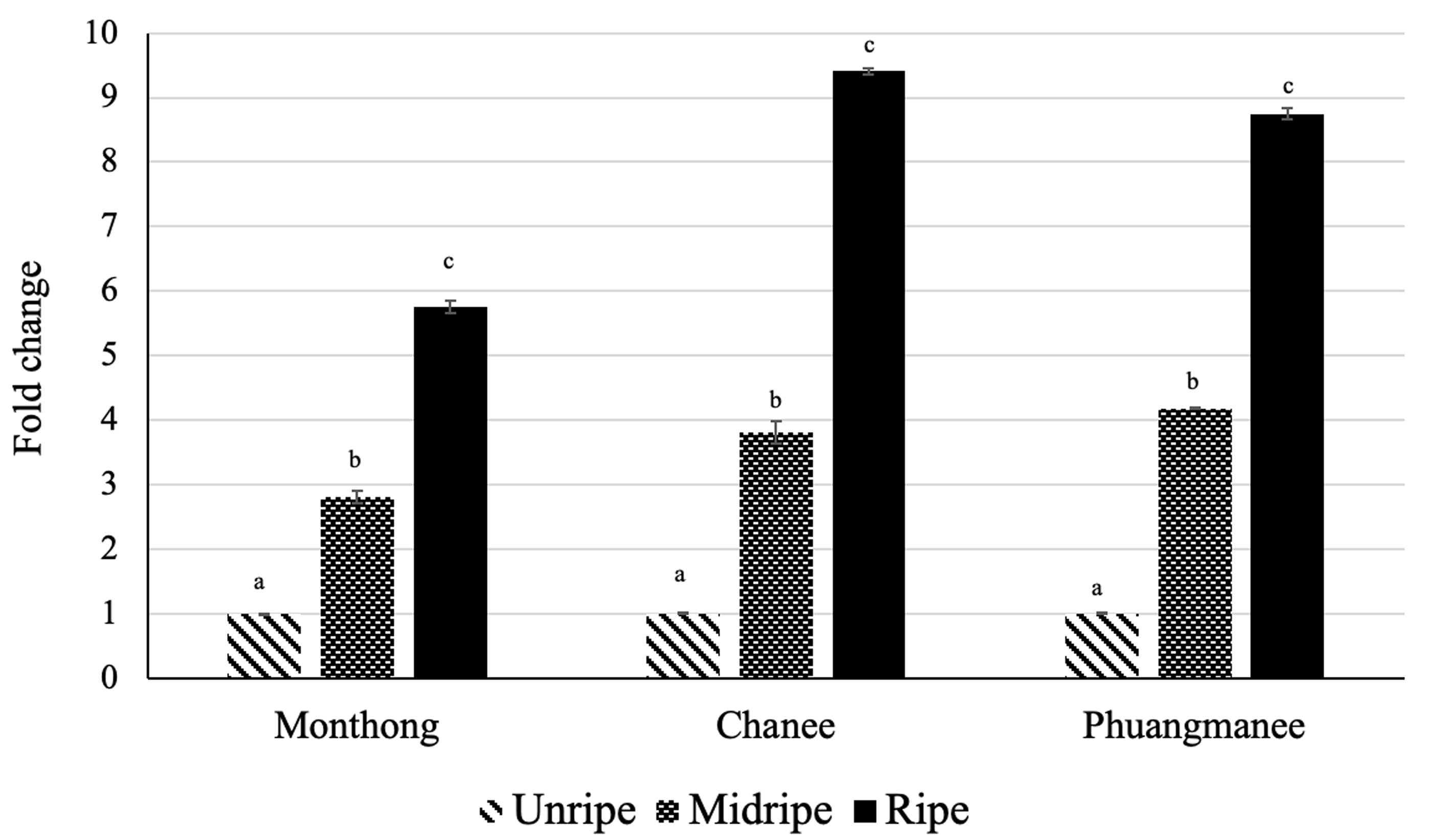
**

**Supplementary Figure S5** Fold changes in expression level of *DzARF2A* during post-harvest ripening of different durian cultivars. The relative expression level of *DzARF2A* was analyzed in fruit pulps of two fast-ripening cultivars (Chanee and Phuangmanee) and a slow-ripening one (Monthong) at three different stages (unripe, midripe, and ripe) during fruit ripening by using the 2^-ΔΔCt^ method and levels were normalized by the geometric mean of reference genes and the unripe stage as control. It should be noted that for Kanyao cultivar, we did not have plant material at unripe and midripe stages for RNA extraction and cDNA synthesis. We included this result as a supplementary figure to confirm the ripening-associated expression pattern of *DzARF2A* in Chanee and Phuangmanee cultivars. Moreover, we wanted to confirm that the expression level of *DzARF2A* is significantly higher not only at ripe stage, but also at other stages in fast-ripening cultivars compared to the slow-ripening one. Three independent biological replicates were used. For each cultivar, bars with different letters show significant differences (*P* < 0.05).

**
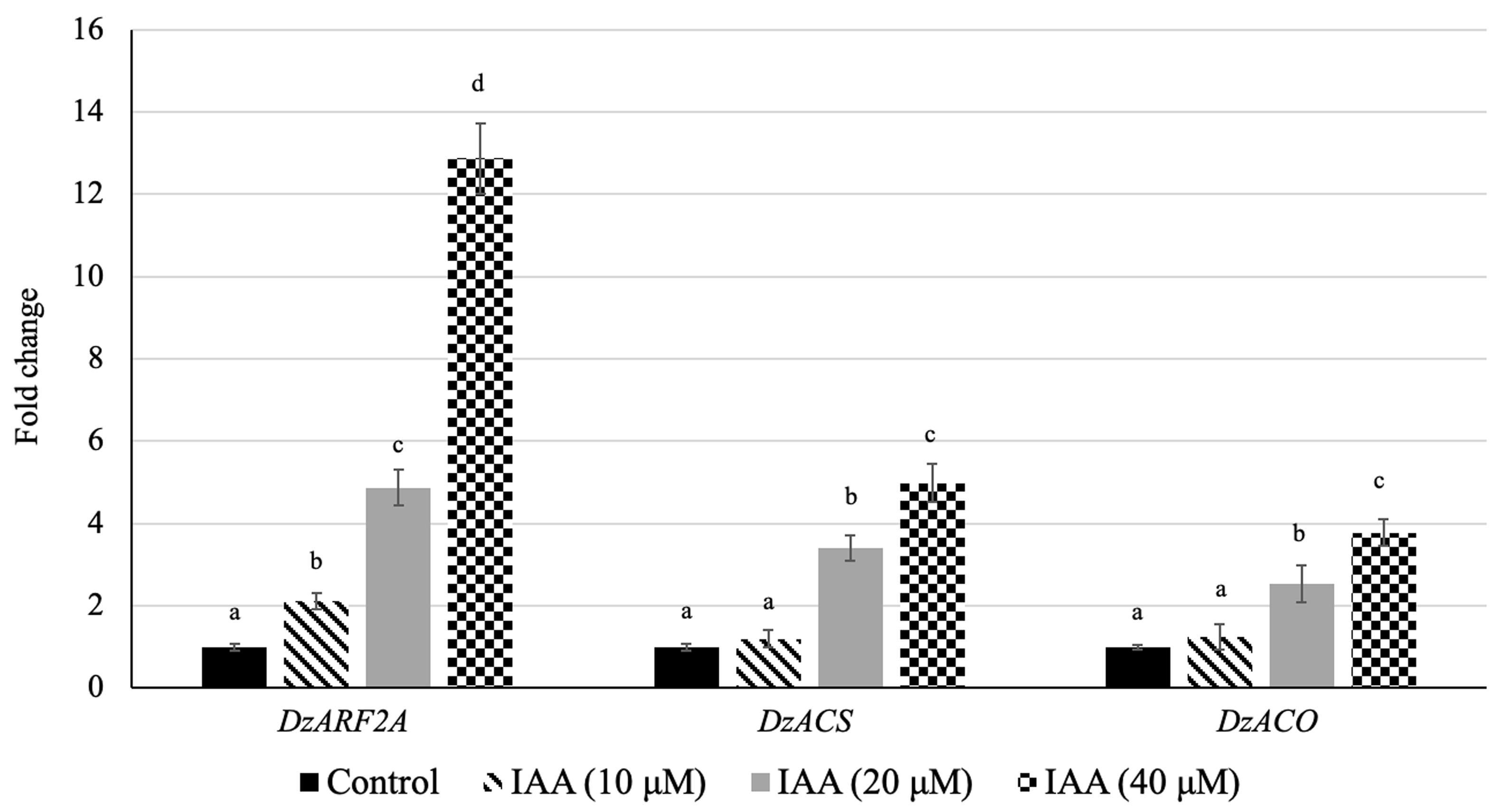
**

**Supplementary Figure S6** Fold changes in expression levels of *DzARF2A* and ethylene biosynthetic genes in durian leaves treated with exogenous auxin. The relative expression levels of *DzARF2A* and ethylene biosynthetic genes of durian; *DzACS* and *DzACO* were measured in the durian leaves treated with increasing concentrations of exogenous auxin by using the 2^-ΔΔCt^ method and levels were normalized by the geometric mean of reference genes and the control samples (0 μM IAA). Three independent biological replicates were used for each condition. Bars with different letters indicate significant differences (*P* < 0.05).

***Durio zibethinus ACC synthase* (*DzACS*)**

CTTTGACCTGCAGAAAATTCTAATAATGCAGTAGATCTTTACGTTGACTTAAGTTTAGGATATAGCTAGC

CAATTGTTTGGACAATTCTCCTCTTACTTGGTTTCCTTATAAAAAGCTCATGTACAACTTGTTCTTTTAT

ATATAGTTAATCTTCTTTTTTCTAATACTCTTCCTATATCAGTTTTAGTCAATTGTTTTTTATTTAAAAA

CTATGCAGATTTGCCCGGGACCTAAGATGGCGCACTGGGTTAGAAATAGTTCCAGTTGACTGCAAAAGCT

CAAACAATTTTCGTATAACAAGAGCAGCCCTGGAAGAAGCCTATGAAAAAGCTCAAAGATCAAACATCAA

TGTCAAAGGCGTGATCATAGCAAACCCCTCAAACCCTTTAGGCACAATCTTGGACAGAGAGACAATGAGA

AGCATAGTCAGTTTTATTAACGAACAGAACATCCACCTTGTCTCCGATGAAATCTATGCAGCTACAGTCT

TCAGCTCTCCTAGATTCATTAGCATTGCTGAGATTATACAAGATATGGATTGTAACCGTGATCTGATTCA

CATTGTTTACAGCTTGTCCAAGGACATGGGATTTCCTGGTTTTCGAGTTGGCATTGTTTACTCATTCAAT

GATGTAGTTGTGAATTGTGCCCGCAAGATGTCAAGTTTCGGATTAGTCTCCTCGCAAACTCAATACTTAC

TTGCTTCAATGCTTTCTGACGAGGATTTTGTTGGGAATTTCCTAAGGGAAAGCTCAAAGAGGTTAGCCAA

ACGGCACCATGTGTTCACCAAGGGACTCGAACAAGTGGGGATTTCTTGTCTAAAAAGCAATGCTGGTTTG

TTTTTCTGGATGGACATGCGACCCCTCCTTAAAGAACAAACTGTCAAGGGAGAAATGGAATTGTGGCGTG

TGATTATCGATGAAGTGAAACTCAATGTTTCTCCAGGTTCATCTTTCCAATGCTTGGAACCTGGCTGGTT

CAGGGTCTGCTTTGCAAACATGGATGGTGAGACCGTGGAAGTAGCACTTGACAGAATTCGAAAATTTGTG

CTTCAAGGAAAGGAAGAAGATCATGCGGTGCCAGAGACGTCAAAACGTTGGCAAAAGAAAAATCTTCGCC

TCAGCTTCTCTTCCTCTAGGTTATACGACGAGAGTATCATGTCTCCACCCATGATTTCCCCTCACTCCCC

GATACCTCACTCGCCCCTCGTTCCGGCGATGACTTGAGTAATAATTACAACGTAGCATTGCATGTCTCAT

CTTCGTAGTTCATCGTATTCATCTTTAATTAGTAATTTAAGTCATAAAAGTTCAATAGGAAATTCATTGT

CATAGATATCATTTTTAAGAGATTATTATGGCTCAGATTAATTTGGTACTTAAATCTTTTTGGTCTGACG

CCGATTTATTAAACCTCTTGATTTAGAGCTTAGGTGGAGAATAATAATCTTTTTCTCTAATTAATTTTTT

CCAAATTATAAAGTCTATGCTGTATGTAAGCATTATTTTTGTGGAGATATGCATTGTACTAGTAGTAATT

GTTCTATTCTAGGAGTCTTAGTTTGTACCAAGATGTTTGATTATAGTAATAATAATAATAAATGTGATTT

AGGATTCTAATTATTTTTATGAATTGAATTTGTGGTGTGCATCTAAAGCTTGAGGTCGCTGTGGAGAACA

CTAAGATTCTCAATTATCTTCATTCTTATGGGTTTTGATTTAACATTTTAATCACTAACTAAAGGTTAGC

TAAGTGGATGCTAAATAATCAATATGACATCATTGGATTGGCAATAACAGTATCCGTGCCTTCAATTTGC

TTTGGTGGAGCAAACATATAAATAAAACAGCTCAGACAAACAACCATGGCATGGAACATGATGTGATCAT

GGCATGTGCATTATGGATAAAACTATATGCAAATTTTCTTGGACATTTGTATATATACCCCATTTGAAAC

AGTGACTCAATGAAATTGAGAAGTTTTCAGGTCATGATCCATG

***Durio zibethinus ACC oxidase* (*DzACO*)**

AAGGCAAAGGCACTTTAGGAAGTCTTTTATGATTTTTGGGTCATCAAAAAGTTGGTCTAATCCCACTCAA

AGAATCCAACCCAGGCCAAAGAGTGGATAGCCACTTCAACAAGGCAAAGGGACTTTAGGAAGTCTTTTAT

GATTTTTGTTTCATTTGCTTTTACTATTATTTTCCCATTTCCTTTTTTTTTTTTTTTGTTAATTTTTAAC

TACATAATTTGCCTGACCCCCAAACCCTCCACTTGGAACTTCTATAAATACCACCACTCCTAGCTTCCAC

TCTGCAATCCGGAAATCTAAACTTTTGCGTAACACCCAAAGCAAGCGAGTGAAGACTTTGAGAGATTTTT

AGGAAAGAAAAAGAAAAGAGTGCTAAGAGAAATGGCAACTTTCCCAGTGATCTACATGGACAAGCTTAAT

GGTGAGGAGAGGGCAGCAACCATGGAGAAAATCAAGGATGCCTCTGAGAACTGGGGCTTCTTTGAGGTAT

CAAAAGGCTTAGCTATGCAGCTATGTACTATACTCTATTCTCCTCTACCTTTCTCCTTTATATTTTACTG

ATACGGTTATTTCTTCATTTCTTCCAGCTGCTGAACCATGGGATTCCCCATGATTTTCTGGACACTGTTG

AAAGATTGACAAAAGAGCATTACAAGAAATGCATGGAGCAGAGGTTTAAGGAACTGGTAGCAAGCAAGGC

CCTGGAGGGTCCCCAGGCAGAGGTGACTGATATGGATTGGGAGAGTACATTCTTCTTGCGCCATCTCCCT

GAATCAAACATGGCTGAAATTCCAGATCTCACTGATGAATACAGGTACAGAAGGAGTAAATTACAAGTTT

CAAAATGAAGTCAGATGTTCTAAATTCAATAAAAATAAATAAATTAGTCCCCTCCTAATGGTGCTTATTT

GGACAACAATAACTTTTCCCACTAAATTTCTTTCCCAGGGCTGTCTCCATAATTGGTTAATGCTGTCTCC

TCTTTCTTGACCAATGAATATAATATTGAATGATTTGACCATCATTGGCTTTTAGAATAATACCAACAAT

TACTTACTGCAATAAATTTTTCCCACTTTGTCTCTGTTTTGAAAACTTGCAAGTCCTGATACTTAGTCCA

AGTAAACAAAAATGCAGGAAGGTGATGAAAGAATTTGTACTGAAATTGGAGAAACTAGCGGAGGAGCTCC

TAGACCTGTTGTGTGAGAACCTTGGACTAGAGCAAGGATACCTGAAAAAGGCCTTCTATGGGGCAAGAGG

TCCAACCTTTGGCTCCAAAGTTAGCAACTACCCACCATGCCCAACCCCAGACAAAATCAAGGGACTCAGA

GCCCATACAGATGCAGGTGGCATCATCCTGCTCTTGCAAGACCCTAAAGTGAGCGGCCTCCAGCTTCTTA

AAGACGGGGAATGGATCGATGTTCCACCCATGCGCCACTCCATTGTAATCAACCTTGGGGATCAGCTCGA

GGTATGCACTCTAAATCTTTACAATTTGCTTTTAGCTCAGTGCTTTTAGAAAGGAAAGTTAGACATCAAA

TTCTAAATCCTTAAACATACAGGTGATCACCAATGGCAAATACAAGAGTGTGGAGCACAGAGTGCTTGCC

CAAACTGACGGAGCTCGCATGTCTCTAGCTTCATTCTACAACCCTGGCAGGGATGCCCTTATCTACCCTG

CACCAGCTCTGGTGGAGAAAGAAGCAGAGGAGAAGAAACAATTGTACCCCAAATTTGTGTTTGAAGACTA

CATGAAGCTTTATGCTGGACTGAAATTCCAGGCCAAGGAACCAAGATTTGAAGCCATGAAAGCCAGGGAA

ACAACTTTTCCCATTGCAACAGCTTAAAATTCTAGAGCTTCGTTTGACTTGATGGAGAAAAGGATGTGAT

CTATTTTCAACCTTTTAGTTGTGTGTGAAAGAAAAAAAAAAGAAGTCAAAGCTTACTGTAGGTGTGTGTT

GATATATTACTGTAAACAGCAACAACAATTCTATTCTACTATG

**Supplementary Figure S7** Nucleotide sequences of promoter regions of ethylene biosynthetic genes from durian (*DzACS* and *DzACO*). ARF binding sites (TGTCTC) are highlighted in yellow. The translational start site (ATG) is underlined.
